# When Lie Groups Meet Hyperspectral Images: Equivariant Manifold Network for Few-Shot HSI Classification

**DOI:** 10.3390/s26072117

**Published:** 2026-03-29

**Authors:** Haolong Ban, Junchao Feng, Zejin Liu, Yue Jiang, Zhenxing Wang, Jialiang Liu, Yaowen Hu, Yuanshan Lin

**Affiliations:** 1 College of Information Engineering, Dalian Ocean University, Dalian 116023, China; 2 Laboratory for Big Data and Decision of NUDT, National University of Defense Technology, Changsha 410004, China; liu_1999@nudt.edu.cn (J.L.); yaowenhu@nudt.edu.cn (Y.H.)

**Keywords:** hyperspectral image classification, few-shot learning, Lie groups, geometric invariance, SE(2) equivariance, affine Lie group, manifold modeling, remote sensing

## Abstract

**Highlights:**

**What are the main findings?**

- We propose EMNet, a Lie-group-guided framework that explicitly encodes geometric invariance for few-shot hyperspectral image classification, consisting of an SE(2)-based equivariance-guided module and an affine Lie-group feature-filtering convolution.
- Experiments on standard datasets (WHU-Hi-HongHu, Houston2013, Indian Pines) and real-world scenes (OHID-1, large-scale Xiongan New Area scene) show EMNet achieves consistent accuracy and robustness improvement over the DGPF-RENet baseline, with up to +3.34% OA, +6.01% AA, +4.14% Kappa under low-shot protocols, and +8.99% OA, +13.25% Kappa under 1% labeled-sample protocol.

**What are the implications of the main findings?**

- The proposed Lie-group-based framework effectively improves robustness to common geometric transformations, reducing feature drift and class confusion in complex agricultural and urban hyperspectral scenes.
- This work validates the strong generalization and scalability of EMNet under pronounced distribution shift and extreme long-tail class imbalance, providing an effective solution for few-shot hyperspectral image classification in real-world scarce-label scenarios.

**Abstract:**

Hyperspectral imagery (HSI) offers rich spectral signatures and fine-grained spatial structures for remote sensing, but practical HSI classification is often constrained by scarce labels and complex geometric disturbances, including translation, rotation, scaling, and shear. Existing deep models are typically developed under Euclidean assumptions and rely on data-hungry training pipelines, which makes them brittle in the few-shot regime. To address this challenge, we propose EMNet, a Lie-group-based Equivariant Manifold Network for few-shot HSI classification that explicitly encodes geometric invariance and improves discriminative accuracy. EMNet couples an SE(2)-based Equivariance-Guided Module (EGM) to enforce equivariance to translations and rotations with an affine Lie-group-based Characteristic Filtering Convolution (CFC) that models scaling and shearing on the feature manifold while adaptively suppressing redundant responses. Extensive experiments on WHU-Hi-HongHu, Houston2013, and Indian Pines demonstrate state-of-the-art performance with competitive complexity, achieving OAs of 95.77% (50 samples/class), 97.37% (50 samples/class), and 96.09% (5% labeled samples), respectively, and yielding up to +3.34% OA, +6.01% AA, and +4.14% Kappa over the strong DGPF-RENet baseline. Under a stricter 25-samples-per-class protocol with 10 repeated random hold-out splits, EMNet consistently improves the mean accuracy while exhibiting lower variance, indicating better stability to sampling uncertainty. On the city-scale Xiongan New Area dataset with extreme long-tail imbalance (1580 × 3750 pixels, 256 bands, and 5.925 M labeled pixels), EMNet further boosts OA from 85.89% to 93.77% under the 1% labeled-sample protocol, highlighting robust generalization for large-area mapping. Beyond point estimates, we report mean ± SD/SE across repeated splits and provide rigorous statistical validation by computing Yule’s Q statistic for class-wise behavior similarity, performing the Friedman test with Nemenyi post hoc comparisons for multi-method ranking significance, and presenting 95% confidence intervals together with Cohen’s d effect sizes to quantify practical improvement.

## 1. Introduction

Hyperspectral imaging (HSI) acquires dense, contiguous spectral bands that encode fine-grained spectral signatures of surface materials while retaining detailed spatial textures and structural patterns. This joint spectral–spatial description makes HSIs indispensable in applications such as agricultural monitoring, mineral and resource exploration, ecological assessment, and urban land-cover mapping. Yet, the high cost of pixel-wise annotation and the severe scarcity of labeled samples largely constrain the generalization and stability of deep learning models in HSI classification [1]. Under small-sample conditions, models are easily affected by issues such as high-dimensional spectral redundancy, skewed sample distributions, and mixed pixels, which can lead to feature drift and unstable discrimination.

From a modeling standpoint, the key challenge is learning features that are robust to rigid transformations (e.g., rotations and translations) when supervision is limited. Without explicit mechanisms for such invariances, the model becomes strongly data-dependent and overly sensitive to variations in object pose. The second challenge stems from more complex affine deformations, including scaling and shearing, which aggravate the same-object spectral inconsistency and cross-class spectral similarity, thereby blurring semantic boundaries and causing pronounced class entanglement in the feature space. Thus, designing models that can robustly handle multi-scale, geometrically complex perturbations under limited annotation emerges as a central scientific problem in small-sample HSI classification [2,3,4]. These challenges are further amplified in large-scale urban scenes such as Xiongan New Area, where millions of pixels, dominant background regions, and long-tail class distributions increase sampling sensitivity and demand robust geometric inductive biases.

In particular, city-scale hyperspectral mapping demands models that remain reliable when the test set contains millions of pixels and when minority categories occupy only a tiny fraction of the scene; therefore, we emphasize not only few-shot learning but also scalability to large datasets and severe imbalance.

Early deep methods primarily adopt 2D and 3D convolutional neural networks (CNNs) to extract spatial structures and spectral textures within local neighborhoods. 3D convolutions can jointly model spectral–spatial interactions at the voxel level, but they are constrained by local receptive fields and substantial computational cost. Consequently, they are ill-suited for explicitly capturing global dependencies over long spectral ranges and large spatial scales, and their training stability and sample efficiency are often unsatisfactory in small-sample regimes [3,5]. To better address long-range dependencies and label scarcity, Transformer-based self-attention models and state space models (SSM/Mamba) have recently been introduced into HSI classification. Transformers are effective at modeling long-range sequence interactions and thus help uncover global cross-band correlations [1]. Mamba further achieves global sequence modeling with linear complexity, mitigating the quadratic cost bottleneck of attention and showing strong potential in high-dimensional HSI applications [3,4]. Building upon these foundations, structured Mamba variants and spectral–spatial collaborative frameworks (e.g., 3D-SS Mamba and structure-aware Mamba) achieve a favorable trade-off between efficiency and global context modeling without compromising coupled spectral–spatial expressiveness, providing a unified route for handling high-dimensional, small-sample, and heterogeneous HSIs [2,3,4]. Complementary lines of research explore lightweight 3D CNNs and transfer learning strategies to enhance data efficiency under limited labels, jointly with Transformer/Mamba paradigms, advancing spectral–spatial joint representation, long-range dependency modeling, and robustness in small-sample settings [5]. In summary, for high-impact applications in resource exploration, precision agriculture, and environmental management, it is of clear theoretical and practical importance to develop spectral–spatial joint models that can learn efficiently, represent accurately, and generalize reliably from limited supervision [1,2,3,4,5,6,7,8].

The evolution of HSI classification in the deep learning era has evolved through multiple paradigms: sequence modeling, convolutional architectures, and, more recently, self-attention. Early works leveraged recurrent neural networks (RNNs) to exploit the sequential nature of spectral bands and capture long-range dependencies. However, their strict reliance on band ordering hampers cross-dataset generalization and limits the modeling of very long-range spectral dependencies [9]. The dominant direction then shifted toward CNN-based methods, where 3D convolutions in the joint spectral–spatial domain significantly improved accuracy and robustness. Hybrid 3D–2D convolutional frameworks with hierarchical feature fusion further showed that judicious dimensional decoupling and cross-layer fusion can produce a desirable balance between parameter efficiency and classification performance [10].

Driven by small-sample and efficiency demands, a range of network compression and lightweight convolutional designs have been developed. For instance, extremely compact networks with parallel shallow branches and non-deep operators seek to achieve competitive performance with very few parameters [11]; deep yet narrow 3D CNNs combined with transfer learning help alleviate label scarcity while maintaining moderate computational cost [12]; and lightweight architectures based on dense connectivity or depthwise separable convolutions consistently reduce redundancy and shorten inference time [13,14]. In parallel, graph-based methods offer a complementary way to exploit sample relations: by constructing dual semantic graphs and propagating spatial–spectral information with graph convolutional networks (GCNs), these approaches improve discriminative power even when labeled samples are extremely limited [15].

The introduction of Transformers has facilitated more systematic modeling of long-range dependencies. In the HSI context, Hyperspectral Image Transformer (HiT), a spectral-adaptive 3D convolutional projection combined with dimension permutation encoding, preserves local spectral–spatial details while enhancing global context modeling [16]. Studies on scalable vision Transformers reveal that excessively deep stacks can suffer from attention collapse, and show that re-attention mechanisms can stabilize deep training, providing guidance for safely deepening models under remote-sensing small-sample settings [17]. To balance accuracy and computational efficiency, lightweight hybrid architectures that integrate convolution and Transformer have emerged as a compelling design: convolutional layers act as early-stage local inductive biases, while self-attention layers focus on mid-to-late global modeling. Extensive empirical results show that these hybrid designs consistently outperform pure CNN or pure Transformer solutions [18]. Additional works study the complementarity of CNNs and Transformers through pooled attention and cross-scale fusion, leading to end-to-end collaborative frameworks [19]. From an engineering perspective, efficient inference on constrained platforms is equally important. Efficient backbones such as LeViT, a lightweight vision transformer architecture, demonstrate the feasibility of jointly optimizing floating-point operations (FLOPs), latency, and accuracy across different hardware platforms, offering practical blueprints for efficient deployment in HSI scenarios [20].

Despite these advances, current 3D CNNs, Transformers, and state space models are largely built upon Euclidean geometric assumptions. This modeling choice ignores the intrinsic manifold structure of HSIs and the geometric consistency of spectral–spatial interactions, limiting robustness to rotations, scale changes, and non-rigid deformations. To tackle this limitation, this work proposes an explicit geometric modeling paradigm that relaxes Euclidean constraints and constructs a hyperspectral classification framework endowed with strict geometric invariance. Specifically, by leveraging group theory and manifold analysis, we introduce representation mechanisms based on the special Euclidean group SE(2) and affine Lie groups to model equivariant feature flows under continuous group actions such as translation–rotation and scaling–shearing. This perspective establishes a unified geometric foundation for small-sample HSI classification and provides rigorous mathematical support for the model designs developed in later sections, resulting in a coherent link between motivation and innovation.

Our main contributions are summarized as follows:

- Problem-driven few-shot framework with hierarchical geometric robustness: Few-shot HSI classification is mainly challenged by (i) rigid pose variations (rotation/translation) under limited supervision and (ii) stronger affine deformations (scaling/shearing) that amplify spectral inconsistency and class entanglement. EMNet addresses both factors in a unified Lie-group framework: it enforces SE(2) equivariance for rigid motions and applies affine-aware feature refinement to accommodate scaling and shearing, yielding more stable and discriminative representations under few-shot supervision.
- First explicit Lie-group geometric paradigm for small-sample HSI: We introduce a Lie-group- and manifold-based mathematical modeling paradigm for few-shot HSI classification, moving beyond purely Euclidean assumptions and offering an interpretable geometric foundation for designing spectral–spatial learning operators.
- Strong empirical evidence on three benchmarks: Extensive experiments on WHU-Hi-HongHu, Houston2013, and Indian Pines under small-sample protocols consistently verify the effectiveness of EMNet, delivering improvements of up to +3.34% OA, +6.01% AA, and +4.14% Kappa over the strong DGPF-RENet baseline. Additional evaluations on OHID-1 and the large-scale Xiongan New Area dataset further confirm cross-scene generalization under strong distribution shift and extreme long-tail class imbalance, with clear gains under the 1% protocol on both datasets (e.g., Xiongan: 85.89% → 93.77%, +7.88% OA).

## 2. Related Work

### 2.1. Mathematical Modeling in Computer Vision: Progress and Limitations

The development of computer vision has long been intertwined with advances in mathematical modeling. Classical methods, grounded in linear algebra and calculus, used convolution, gradient operators, and Fourier analysis to transform images and extract low-level features. As probability and optimization theory were incorporated into visual models, representations shifted from pixel-centric processing to describing statistical distributions and underlying geometric structures [21].

Within this trajectory, optimal transport (OT) has become an important tool in graphics and vision, providing principled formulations for shape matching, density estimation, and geometric alignment. Its emphasis on global consistency and cross-domain mappings illustrates the unique strengths of mathematical models for high-level visual reasoning [22]. These contributions form the mathematical backbone of contemporary computer vision and support high-dimensional distribution modeling and metric learning.

Beyond low-dimensional formulations, recent studies have pushed vision problems toward cross-modal and logical reasoning. For example, the MATHVERSE benchmark adopts a multimodal chain-of-thought setting to test whether multimodal large language models (LLMs) can sustain coherent and logically consistent visual–mathematical reasoning [23]. MathVision further unifies OCR, visual understanding, and logical calculation, offering a comprehensive testbed for evaluating multimodal mathematical reasoning [24]. Together, these efforts suggest that mathematical modeling in vision is shifting from purely geometric formulations toward integrated multimodal reasoning.

Deep learning has catalyzed a new stage of interaction between mathematics and vision. convolutional neural networks (CNNs), built on local connectivity and parameter sharing, remain the workhorses of image recognition and classification. Reviews indicate that architectures such as U-Net and generative adversarial networks (GANs) deliver strong performance in medical image segmentation, organ recognition, and tissue classification, and can be interpreted as high-dimensional approximators of nonlinear operators [25]. In endodontics, artificial neural networks (ANNs) optimized with carefully designed loss functions achieve high-precision lesion detection and prognosis prediction [26]. Parallel studies on CNN interpretability, particularly layer-wise visualization in radiology and pathology, have improved transparency and offered quantifiable support for clinical decision-making and training [27].

Mathematical modeling also supports a wide range of application domains. Autonomous driving systems integrate deep CNN-based perception with decision-making and control modules into a unified pipeline [28]. Precision agriculture combines multispectral sensing with vision–language models to enhance spatiotemporal representations and classification of plant diseases [29]. Assistive systems for visually impaired users re-encode visual input into semantic descriptions through deep semantic modeling and interactive interfaces [30]. Large-scale urban scene understanding, supported by semantic segmentation, provides structured representations for city-level perception and navigation [31]. Furthermore, robot control in dynamic environments benefits from the joint use of reinforcement learning and visual perception to enable image-driven autonomous decision-making [32].

Despite their success in standard vision tasks, Euclidean-space-based models and deep convolutional neural networks (DCNNs) face fundamental challenges when applied to hyperspectral images. HSIs are high-dimensional and exhibit strongly coupled spectral–spatial structure. Conventional designs usually rely on fixed Euclidean embeddings and metrics, which makes it difficult to explicitly characterize geometric consistency under transformations such as rotation, translation, scaling, and shear.

To address this limitation, we introduce an explicit geometric modeling framework. Specifically, we incorporate the special Euclidean group SE(2) and an affine Lie-group-based operator into the network to regularize intermediate spectral–spatial representations under continuous geometric actions. In this sense, the proposed method does not perform nonlinear manifold learning on the raw input; rather, it imposes a geometry-aware structure on the learned feature space after standard spectral compression.

### 2.2. Hyperspectral Image Classification

Hyperspectral image classification targets accurate pixel-wise recognition of land-cover categories by exploiting joint spectral–spatial information, and is central to intelligent remote sensing analysis. With the emergence of deep learning, HSI classification has progressed from conventional convolutional architectures to Transformer-based and state-space models [33].

Representative convolutional models include DCN-T, which adopts a dual-context design and triple-spectral generation modules to fuse local and global spatial information while improving structural interpretability [34]. AFLA-SCNN introduces an adaptive Fick’s law-based spectral diffusion mechanism for dynamically modulating spectral responses, enhancing robustness to complex spectral variations [35]. Wav-KAN embeds wavelet kernels into a Kolmogorov–Arnold network to capture stable spectral–texture relations across multiple scales [36].

To overcome CNNs’ limitations in modeling long-range dependencies, Transformer-based architectures have been proposed. Masked-ViT uses self-supervised masked reconstruction to learn spectral–spatial correspondences from limited labeled data [37], while SS-MTr couples supervised and contrastive objectives to enforce semantic consistency and reconstruction across spectral and spatial branches [38].

State-space models offer more efficient global modeling. SS-Mamba performs linear-complexity modeling of long-range dependencies, and MorpMamba further introduces morphological operators to better handle complex land-cover geometries [39,40].

In cross-domain and data-scarce scenarios, multimodal and generative approaches have gained momentum. LDGnet introduces vision–language alignment into HSI classification and exploits language priors for cross-scene semantic generalization [41]. SDEnet employs single-source domain expansion to diversify feature distributions and strengthen domain adaptation [42]. SpectralDiff leverages diffusion-based generative modeling to characterize dynamics in the spectral domain through bidirectional denoising, enabling self-consistent spectral–spatial reconstruction [43]. These methods show that combining generative models, contrastive learning, and state-space architectures is a promising direction for mitigating small-sample constraints.

For spatial structure modeling, coupling regional interactions with attention has proven effective. CSIL formulates a center-to-surrounding interactive learning framework, where a hierarchical region sampling strategy explicitly generates multi-level regions (center/neighbor/surrounding) and feeds them into a center transformer and a surrounding transformer for interactive fusion of fine-grained details and coarse contextual cues [44]. PSANet employs dual-branch polarized self-attention to enhance joint spectral–spatial encoding [45].

In remote sensing object detection, edge-aware priors have been explicitly integrated to cope with challenging weather and heavy noise. RSFC-EAFANet combines robust scale-fusion convolution with an edge-aware attention module to emphasize road boundaries and suppress weather noise, significantly improving detection robustness in adverse conditions [46]. PSWP-DETR, built on a Transformer backbone, fuses adaptive deformation learning with multi-scale feature integration: PdConv models horizontal and vertical geometric deformations, and a scale-difference module combining multi-scale and edge-capturing convolutions aggregates multi-scale and boundary texture cues, greatly improving detection of small objects with complex scales in remote sensing imagery [47].

For extreme lightweight and binary networks, novel convolutional variants have been proposed. HBiPiDiNet uses binary pixel-difference convolutions (BiPDCs) and heterogeneous kernel fusion blocks (HKFCBs) to strengthen local contrast priors and multiscale responses under fully binarized weights, yielding clear gains in small-object detection with complex scale distributions [48]. These techniques typically rely on explicit feature fusion or implicit alignment to enhance stability and discriminability in challenging scenes.

Very recently, several strong designs have further advanced hyperspectral analysis under limited supervision. Zhang et al. propose PFS3F, which probabilistically fuses superpixel-wise spatial cues with semantic-aware structural features, thereby mitigating boundary inconsistency caused by imperfect segmentation [49]. Liang et al. introduce LKMA to combine learnable-kernel multiscale feature generation with a Mamba-based global mixer, and use spatial–spectral attention fusion to balance local detail and global context at linear complexity [50]. Along a similar line, DBMLLA adopts a double-branch Mamba-like linear attention design to model spatial and spectral dependencies efficiently, with depthwise separable convolutions and feature fusion to enhance local discrimination [51]. For fast inference, FTSCN integrates a squeezed-enhanced axial Transformer with SimAM-guided CNNs and dual attention units to capture both long-range spectral relations and hierarchical spatial textures [52]. Beyond classifiers, MSGHSU introduces a multiscale framelet decomposition together with adaptive superpixel-graph regularization to improve robustness to noise and mixed pixels, a persistent source of confusion in HSI interpretation [53]. Earlier, MDRN couples a multiscale DenseNet with an attention-equipped Bi-RNN to jointly exploit multiscale spatial structures and band-wise spectral correlations [54]. CAEEFT-Net further strengthens boundary delineation through cross-stage attention edge enhancement and integrates Fourier–wavelet Transformer blocks for frequency-aware global modeling [55].

Building on these advances, ST-GGNet by Li et al. introduces a rank-aware “gaze–glance” attention mechanism and texture enhancement modules to jointly model long-range spectral dependencies and fine-grained textures in HSIs. Together with a bud-growth optimization scheme, ST-GGNet consistently outperforms multiple SOTA baselines on several public HSI datasets under low-sample regimes, highlighting the importance of explicitly modeling spectral–spatial long-range relations and texture details for robust small-sample HSI classification [56].

In parallel, foundation-model research is emerging for hyperspectral understanding. HyperSIGMA unifies HSI interpretation across tasks and scenes through large-scale pretraining, introducing sparse sampling attention to reduce spectral–spatial redundancy and a spectral enhancement module to better integrate spatial and spectral cues [57].

At the architecture level, SpectralFormer revisits HSI classification from a sequential perspective by learning groupwise spectral embeddings from neighboring bands and preserving informative components through cross-layer skip connections [58]. For CNN-based multiscale modeling, EMFFN extracts complementary spectral and spatial features through cascaded dilated spectral convolutions and parallel multipath spatial branches, followed by hierarchical feature fusion for improved continuity across scales [59]. For cross-scene settings with limited labels, DHSNet adopts a dual-head self-training strategy with class-wise feature alignment and central feature attention, helping mitigate domain gaps while suppressing the accumulation of erroneous pseudo-labels [60].

Existing HSI classification methods, ranging from 3D CNNs to Transformers and recent Mamba-style models, achieve strong performance by implicitly learning nonlinear spectral–spatial mappings through sophisticated architectures. However, their theoretical foundations remain essentially Euclidean, so they do not explicitly model the high-dimensional spectral–spatial structure and geometric consistency of hyperspectral features under complex transformations. This limitation can manifest as instability under object deformation, scale changes, and mixed noise. In addition, many methods depend on heuristic architectural refinements and do not systematically encode sparsity and geometric constraints, making them prone to decision ambiguity and feature drift in small-sample settings.

Group-theoretic equivariant learning has also been explored in general computer vision. For example, Group Equivariant Convolutional Networks (G-CNNs) replace standard planar convolutions with discrete group convolutions so that the whole backbone becomes equivariant to transformations such as translations, rotations, and reflections [61]. Our work is related in spirit but differs substantially in scope and objective. Rather than redesigning the entire network for generic image recognition, we target few-shot HSI classification and focus on preserving local spectral–spatial texture consistency under scarce supervision. Technically, EMNet uses continuous Lie-algebra-driven SE(2) feature alignment and affine-aware filtering embedded at selected stages of an HSI backbone, together with adaptive sparsification to suppress redundant responses and mixed-pixel interference. Therefore, the contribution of this paper is not the first use of group theory in vision, but a task-specific Lie-group/manifold formulation for few-shot HSI classification.

In this work, we instead start from mathematical principles and introduce explicit geometric modeling into the joint spatial–spectral domain through differentiable group actions and adaptive regularization. This framework enforces equivariance and energy stability under rotation, translation, and scale transformations. Grounded in group theory and manifold analysis rather than empirical stacking of modules, it offers a unified and physically consistent representation for HSI classification, and provides the theoretical basis for the geometric-invariance feature extraction and adaptive sparsity mechanisms developed in the remainder of the paper.

## 3. Methods

### 3.1. Overview of EMNet

Few-shot hyperspectral image (HSI) classification is dominated by two persistent factors: the scarcity of labeled samples and the strong geometric variability of land-cover patterns caused by viewpoint, terrain, and intra-class diversity. EMNet is designed around an explicit symmetry-aware principle: instead of relying on implicit robustness, it embeds Lie-group equivariance into the feature extraction process so that the representation changes predictably under common spatial transformations.

Given an input feature cube (after a lightweight spectral compression step such as PCA), EMNet builds two complementary pathways. In the implementation used in this paper, EMNet is instantiated as a lightweight enhancement of the baseline HSI backbone rather than as a completely standalone architecture. The input HSI cube is first reduced by PCA and then fed to the spectral–spatial backbone. The proposed modules are introduced to regularize intermediate representations instead of replacing the original spectral modeling path: EGM is used to stabilize local rigid-motion responses in feature space, while CFC performs affine-aware filtering on intermediate features and suppresses redundant activations. As a result, spectral semantics are preserved by the underlying spectral–spatial pathway and the attention-guided semantic stream, whereas Lie-group modeling improves the geometric stability of these spectral–spatial features under few-shot supervision. The geometric stream focuses on preserving transformation-consistent structure, while the semantic stream emphasizes class-discriminative responses. Their outputs are fused to form a robust representation that remains stable under pose changes yet still captures fine-grained material differences. In this work, PCA is used only as a preprocessing step to reduce spectral redundancy and computational burden. The retained spectral dimension is denoted by num_PC and is specified in the experimental settings for each dataset.

Concretely, EMNet is composed of an Equivariance-guided Module (EGM) and a Characteristic Filtering Convolution (CFC). EGM aligns features by generating transformation-aware sampling grids from the SE(2) Lie algebra, improving sensitivity to translation and rotation while avoiding brittle hand-crafted alignment. CFC extends this idea to more general affine distortions and introduces a saliency-driven sparsification that suppresses noisy or redundant activations on the feature manifold.

**Figure 1:**
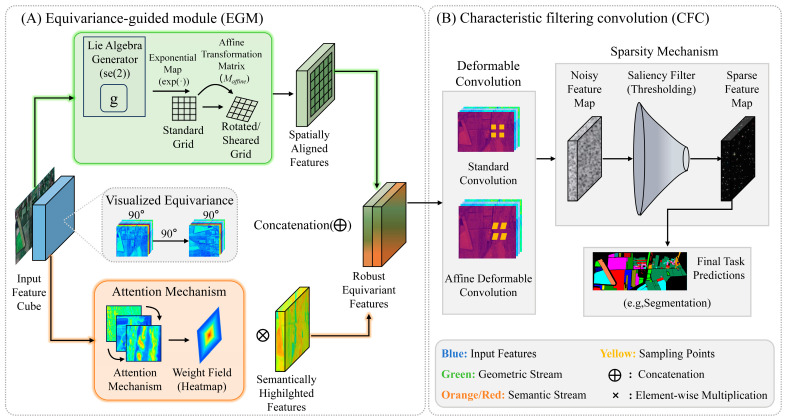
Overall architecture of EMNet: (**A**) Equivariance-guided module (EGM) that produces spatially aligned, equivariant features with an attention-based semantic weighting; (**B**) Characteristic filtering convolution (CFC) built on affine deformable convolution with a sparsity mechanism.

These modules are implemented as convolution-friendly operators with shared parameters and local computations. As a result, EMNet scales well to large scenes: the additional cost grows approximately linearly with the number of pixels, which is important for high-resolution HSI benchmarks with millions of labeled samples and long-tailed class distributions. For the specific content, please refer to Figure 1.

In Figure 1A, the Lie algebra generator g in SE(2) is mapped through the exponential map to obtain an affine transformation matrix, which converts a standard grid into a rotated/sheared sampling grid and yields spatially aligned features. An attention branch produces a weight field (heatmap) to highlight semantically important responses; element-wise multiplication with the aligned features refines this emphasis, and concatenation fuses the geometric (green) and semantic (orange/red) streams into robust equivariant features. In Figure 1B, CFC replaces standard convolution with affine deformable convolution to accommodate more general spatial deformations. A sparsity mechanism further filters a noisy feature map via a saliency thresholding operation, producing a sparse feature map that improves downstream predictions (e.g., segmentation). Blue denotes input features and yellow indicates sampling points.

For clarity, Table 1 summarizes the instantiated layers and tensor notations of the newly introduced modules in Figure 1. Here, H, W, and C denote the spatial height, width, and channel number of the current feature map, respectively, while num_PC denotes the PCA-reduced spectral dimension in the 19 × 19 patch-based implementation. The EGM branch consists of SE2Transform, AttmFuse, and SE2GraphFusion, whereas the affine Lie-group operator is inserted at the second spectral Conv1D stage of the CFC branch.

Although EMNet is developed and validated for few-shot HSI classification, the two proposed modules are not tied to HSI sensing physics alone. Because they operate on intermediate feature tensors and local geometric transformations, they are, in principle, transferable to multispectral or RGB imagery. Nevertheless, HSI remains the primary target of this study, since dense contiguous bands, spectral redundancy, and mixed-pixel effects make the benefit of explicit geometric regularization especially pronounced. Accordingly, we do not claim equal performance on multispectral or color images without dedicated experiments.

### 3.2. Equivariance-Guided Module

The equivariance-guided module (EGM) is built on the symmetry principle: HSI features should transform equivariantly under spatial rigid motions. We model the HSI feature field as a tensor field on a 2D spatial manifold and implement an EGM based on the special Euclidean group SE(2). Standard DCNNs do not explicitly represent such continuous geometric symmetries and thus are fragile to translations and rotations. Introducing SE(2)-equivariant operators enables explicit control of the intrinsic geometry of the feature manifold. Because the SE(2) transform is realized through differentiable grid sampling with only a few learnable parameters, it introduces negligible parameter overhead and can be applied to large feature maps in city-scale scenes such as Xiongan New Area.

Here, the group is the isometry group of the plane, defined as the semi-direct product of the rotation group SO(2) and planar translations. As a three-dimensional Lie group, its geometric behavior is determined by the Lie algebra SE(2). Any transformation can be expressed by exponentiating the Lie algebra basis {*T*_x_, *T*_y_, *T*_z_} through exp(·), corresponding to infinitesimal actions on the feature manifold.

(1) $$M(\theta ,t_{x},t_{y})=\left(\begin{array}{ccc} \cos\left(\theta \right) & -\sin\left(\theta \right) & t_{x}\\ \sin\left(\theta \right) & \cos\left(\theta \right) & t_{y} \end{array}\right)\in R^{2\times 3}$$

The SE2Transform layer learns differentiable parameters of these actions. It maintains a trainable rotation angle and a translation vector *t* = (*t*_x_, *t*_y_), where *t*_x_ and *t*_y_ are translations along the x- and y-directions. These parameters are assembled into a 2 × 3 affine transformation matrix *M* applied to the feature maps, as defined in Equation (1).

(2) F(g·x)=D(g)F(x)

The learned affine matrix is expanded over the batch axis to obtain M_batch_, and applied to the feature tensors through a spatial sampling operator T. This operation produces a geometrically stable feature stream and enforces the equivariance condition of the feature field under SE(2) transformations, as stated in Equation (2). In this formulation, $\rho_{SE(2)}$ denotes the SE(2) representation acting on the feature space R^C^.

In Equation (2), *g* ∈ SE(2) denotes a planar rigid-motion element and *g*·*x* is its action on the spatial coordinate *x*. We use *D*(*g*) to denote the corresponding feature-space representation of *g*.

(3) $$A_{weight}=\sigma ({Conv}_{1\times 1}(A_{1}⊗A_{2}⊗A_{3}))$$

The SE2GraphFusion module jointly encodes geometric and semantic information in a unified tensor-field view. On the semantic side, it first builds a semantic flow: three attention maps $A_{1},A_{2},A_{3}$ are merged by the AttmFuse (Conv2D) block to form a weight field $A_{weight}$ (Equation (3)), which realizes graph-guided aggregation and semantic similarity modeling. The original features X are then modulated by $A_{weight}$ via element-wise multiplication, resulting in a guided semantic stream and a semantically enhanced feature field, i.e., an adaptive semantic filter over the field.

Here, ⊗ denotes element-wise multiplication, Conv_1 × 1_(·) is a 1 × 1 convolution, and σ(·) is the sigmoid function.

(4) $$G_{1}={Conv}_{1\times 1}(X_{guided}⨁T_{M}(X))\in R^{B\times H\times W\times C_{fuse}}$$

In parallel, a spatial encoding flow captures explicit rotations and translations of the feature maps. Its action is defined by SE(2)-equivariant operators, with the special Euclidean group SE(2) providing the theoretical backbone for equivariance coding. The outputs of the semantic and geometric flows, denoted $X_{sem}$ and $X_{geo}$, are finally concatenated along the channel dimension and projected to a lower-dimensional space, completing the cooperative coupling and dimensionality reduction of the feature tensor field, as formalized in Equation (4).

In Equation (4), ⨁ indicates channel-wise concatenation, and T_M_(X) denotes the feature map warped by M through the sampling operator T.

This joint-field fusion implements a principled geometric decoupling: spatial pose, regulated by SE(2), and spectral semantics, governed by the attention-based weight field, are modeled independently yet fused coherently. As a result, the representation attains improved stability and discriminative power.

### 3.3. Characteristic Filtering Convolution

Region-centric modeling has shown that explicitly separating the center from its surrounding context can strengthen multi-scale reasoning in hyperspectral imagery. For example, Yang et al. propose a center-to-surrounding interactive learning (CSIL) framework, where a hierarchical region sampling strategy first generates center, neighbor, and surrounding regions (after PCA-based band reduction), and then uses a center transformer and a surrounding transformer to interactively fuse fine-grained details with coarse contextual distributions [43].

Inspired by this center-to-surrounding perspective, our CFC does not replicate their input-space sampling/tokenization. Instead, it revisits “multi-granularity” from a different angle: we enforce geometric consistency inside the network by learning continuous group actions and using them to filter intermediate feature maps. Concretely, CFC parameterizes affine transformations in the Lie algebra, deforms the convolutional sampling grid accordingly (Equation (10)), and couples this deformation-aware extraction with an adaptive sparsification mechanism (Equations (12) and (13)) to suppress redundant responses and mixed-pixel interference. Therefore, compared with hierarchical region sampling that organizes multi-scale regions before feature extraction, CFC acts as a feature-space operator that explicitly targets affine-equivariant filtering and redundancy control under few-shot supervision.

In short, CSIL improves multi-scale context utilization through an explicit region sampling strategy and a Transformer-based interaction pipeline, whereas CFC provides an explicit, continuous equivariance prior and feature-level redundancy suppression driven by affine Lie-group modeling.

To address the pervasive geometric distortions in remote sensing imagery, we introduce an affine Lie-group-based characteristic filtering convolution (CFC), which integrates SO(2)-equivariant convolution with hierarchical feature fusion. The key innovation lies in embedding rotational symmetry into deep networks via carefully designed steerable filters, enabling the model to inherently capture and exploit rotational invariance. Unlike global self-attention, whose cost can grow quadratically with pixel count, the proposed affine-grid deformation and adaptive sparsification operate locally on feature neighborhoods, which keeps the method practical for million-pixel scenes such as Xiongan New Area while still capturing complex scaling and shearing patterns.

(5) $$\left[f*\psi ](g)=\sum_{x\in Z^{2}}f(x)\psi (g^{-1}x)dx,\forall g\in SO(2\right)$$

The method is grounded in the special orthogonal group SO(2). Given an input feature map $Z^{2}\to R^{Cf}$: on a 2D discrete lattice, the group convolution is defined as in Equation (5).

In Equation (5), *f*(*x*) is the input feature at lattice location *x* ∈ Z^2^, and *g*^−1^*x* denotes the rotated coordinate under *g*^−1^; *dx* represents the discrete measure on the lattice and is absorbed into the summation in implementation.

(6) $$\psi (r_{0})=\sum_{k\in Z}D_{k}(\theta )W_{k},D_{k}(\theta )=e^{ik\theta }$$

In this definition, ψ denotes a steerable filter. Rather than being fixed kernels, these filters are constructed via the irreducible unitary representations of the rotation group, as expressed in Equation (6).

In Equation (6), *k* ∈ Z indexes the Fourier modes of SO(2). The term *D*_k_(θ) = e^ikθ^ denotes the *k*-th irreducible representation, and *W*_k_ are learnable coefficients. Together they form a steerable filter whose response varies with the rotation angle θ. The direction *r*_0_ sets the reference orientation.

(7) $$\rho_{out}(g)[f*\psi ]=[\rho_{in}(g)f]*\psi$$

Specifically, $\rho_{l}$ is the *l-th* irreducible representation of SO(2), describing a basic rotational mode, and *a_l_* is its learnable coefficient. This representation-theoretic formulation ensures that the layer is strictly equivariant: rotating the input yields a correspondingly rotated output feature, as required by Equation (7), where $\rho_{in}$ and $\rho_{out}$ are the group actions on the input and output feature spaces.

(8) $$log(g)=\left[\begin{array}{cc} A & t\\ 0 & 0 \end{array}\right]\in ga(2),A\in gl(2,R)$$

Nonetheless, modeling only rotations is inadequate in realistic remote sensing settings, where images are subject to scaling, shear, and translation. We therefore generalize the framework from SO(2) to the affine Lie group $GA(2)=R^{2}⋊GL(2,R)$, and parameterize its affine Lie algebra to capture the full space of affine transformations. The Lie algebra decomposes into a linear component A and a translation vector t, as in Equation (8). Here, ga(2) and gl(2,R) denote the Lie algebras of GA(2) and GL(2,R), respectively.

(9) $$g=exp\left(\left[\begin{array}{cc} A & t\\ 0 & 0 \end{array}\right]\right)$$

The matrix A accounts for rotation, scaling, and shear, while t encodes translations. Any affine transformation is then obtained via the exponential map from the Lie algebra to the group, as shown in Equation (9).

(10) $$F_{out}(x)=\sum_{y\in \Omega }W_{in}(y)K({\phi_{g}}^{-1}(x)-y)$$

On top of this affine Lie-group theory, we define a geometrically equivariant convolution (GEC). Rather than convolving over a static grid, GEC operates on dynamically generated sampling grids that are obtained by applying the learned affine transformations. This yields affine-equivariant feature extraction, with the explicit formulation given in Equation (10).

In Equation (10), Ω denotes the local sampling neighborhood and W_in_(y) is the input feature at position y. K(·) is the kernel function, and ${\phi_{g}}^{-1}$ maps an output location back to the input grid under the learned affine transform g.

The associated transformation field is predicted from learned Lie-algebra parameters, enabling the convolution kernels to adaptively deform in response to local geometric variations.

(11) $$Attention(Q,K,V)=Softmax\left(\frac{QK^{T}}{\sqrt{d}}+logS\right)V$$

To handle geometric variability at the attention level, we introduce a group-equivariant attention mechanism. It augments the standard attention matrix with an additive spatial prior, as in Equation (11).

In Equation (11), *Q*, *K*, and *V* are the query, key, and value matrices, *d* is the key dimension for scaling, and log *S* is an additive spatial prior derived from Lie-algebra coordinates.

This prior encodes Lie-algebra coordinates and acts as a geometric bias, constraining the attention to discover consistent correspondences under rotational symmetry.

(12) $$\frac{\partial \Psi }{\partial t}={div}_{g}(\nabla_{g}\Psi )-k\Psi (1-\Psi )$$

Complementing these equivariant operators, we design a sparse de-redundancy mechanism to distill the representation. We define a saliency function s on the feature manifold and assume that its temporal evolution is governed by a parabolic PDE (Equation (12)), where a positive constant controls the sharpness of saliency boundaries. Here, ∇_g_ and div_g_ denote the Riemannian gradient and divergence on the manifold M.

(13) $$\int_{\left\{\Psi \geq \tau \right\}}dV_{g}=\lambda \cdot Vol(M)$$

The steady-state solution produces a stable saliency distribution, from which an adaptive threshold τ is derived via the integral constraint in Equation (13). In Equation (13), *dV*_g_ is the volume element, Vol(*M*) is the total manifold volume, and λ ∈ (0,1) specifies the retained fraction used to determine τ.

This constraint enforces the volume of the high-saliency region to occupy a fixed fraction of the manifold volume. Consequently, the model automatically keeps the most salient features while discarding redundant ones, achieving adaptive sparsification.

## 4. Experiments

### 4.1. Experimental Environment

This study evaluates Lie-group-based, geometrically invariant feature extraction for few-shot hyperspectral image (HSI) classification under a unified high-performance computing environment to ensure that the reported results are reliable and reproducible.

The detailed hardware and software configurations are summarized in Table 2. On the hardware side, all experiments are conducted on the AutoDL server platform equipped with an Intel(R) Xeon(R) Platinum 8255C CPU @ 2.50 GHz and 40 GB of RAM. The deep models—particularly modules involving Lie-group operations (e.g., SE(2) and affine groups) and high-dimensional tensor processing—are accelerated by an NVIDIA GeForce RTX 2080 Ti GPU with 11 GB of memory.

On the software side, we use Linux as the operating system and Python 3.6.5 as the programming language. The models are implemented in TensorFlow 1.10.0 with CUDA 9.2 support, and trained through the Keras 2.2.0 interface. This setup provides stable training and sufficient parallel computing capacity, forming a solid basis for validating the proposed geometry-invariant feature extraction scheme, which departs from conventional Euclidean assumptions through explicit Lie-group-based modeling.

For clarity, the key implementation details are further summarized in Table 3. The table reports the instantiated backbone, the training strategy, the spatial patch configuration, and the main hyperparameters. In the implementation, the backbone is denoted as model.demo, num_PC denotes the dataset-dependent number of principal components after PCA, disjoint is set to False, only_draw_label is set to True, output_map is set to True, and GPU memory is controlled by setting per_process_gpu_memory_fraction to 0.4 with allow_growth enabled.

### 4.2. Data Description

We conduct experiments on three benchmark hyperspectral datasets: WHU-Hi-HongHu [62], Houston2013 [63], and Indian Pines [64]. The class labels and the number of samples per class are summarized in Table 2. To assess cross-scene scalability and robustness under stronger distribution shift and severe long-tail imbalance, we additionally include OHID-1 and the large-scale Xiongan New Area dataset in separate generalization experiments.

Note that these public benchmarks are single-date acquisitions. Therefore, our statistical evaluation primarily quantifies sampling uncertainty induced by random train/test splits (mean ± SD over 10 runs), rather than temporal variability caused by different seasons or weather conditions. We discuss this limitation and consider cross-season/cross-condition evaluation as future work.

#### 4.2.1. WHU-Hi-HongHu

The WHU-Hi-HongHu dataset was acquired over Honghu, Hubei Province, China, on 20 November 2017 between 16:23 and 17:37 local time. The data were collected by a Headwall Nano hyperspectral imaging sensor (Headwall Photonics, Bolton, MA, USA) with a 17 mm focal length mounted on a DJI Matrice 600 Pro UAV platform (SZ DJI Technology Co., Ltd., Shenzhen, China). During acquisition, the weather was cloudy, the temperature was around 8 °C, and the relative humidity was approximately 55%. The scene is a complex agricultural area with diverse crop types and multiple cultivars of the same crop (e.g., Chinese cabbage vs. baby Chinese cabbage, and different varieties within the Brassica genus).

The UAV flew at an altitude of 100 m. The image has a spatial size of 940 × 475 pixels with 270 spectral bands covering 400–1000 nm, and a ground sampling distance of about 0.043 m.

The dataset contains 22 land-cover classes with the following pixel counts: red roof (14,041), road (3512), bare soil (21,821), cotton (163,285), cotton stalks (6218), rapeseed (44,557), Chinese cabbage (24,103), baby Chinese cabbage (4054), cabbage (10,819), preserved mustard (12,394), Brassica (11,015), small-leaf Brassica (8954), crown daisy (22,507), lettuce (7356), asparagus lettuce (1002), film-covered sow thistle (7262), sow thistle (3010), carrot (3217), white radish (8712), garlic sprout (3486), broad bean (1328), and trees (4040).

The entire image contains 446,500 pixels, of which 386,693 are land-cover pixels (86.59%) and 59,807 are background pixels (13.41%). The class distribution is highly imbalanced: the largest class (cotton) has 163,285 pixels, accounting for 42.25% of all land-cover pixels, whereas the smallest class (broad bean) has only 1328 pixels, yielding a disparity of roughly 123×. Together with pronounced spectral confusion among certain crops, this makes WHU-Hi-HongHu a challenging and representative benchmark for evaluating the robustness of HSI classification algorithms under severe class imbalance.

#### 4.2.2. Houston2013

The Houston2013 dataset was acquired with an ITRES CASI-1500 sensor (ITRES Research Limited, Calgary, AB, Canada) and released as part of the 2013 IEEE GRSS Data Fusion Contest. The image size is 349 × 1905 pixels, comprising 144 spectral bands spanning 364–1046 nm, with a spatial resolution of approximately 2.5 m.

The dataset includes 15 urban land-cover categories with the following numbers of labeled pixels: healthy grass (1251), stressed grass (1254), synthetic grass (697), trees (1244), soil (1242), water (325), residential (1268), commercial (1244), road (1252), highway (1227), railway (1235), parking lot 1 (1233), parking lot 2 (469), tennis court (428), and running track (660).

In total, the image contains 664,845 pixels, among which 15,029 pixels (2.26%) are labeled land-cover samples and 649,816 pixels (97.74%) correspond to background. With relatively high spatial resolution and a reasonably balanced distribution across urban classes, Houston2013 [63] provides a solid benchmark for hyperspectral urban scene classification.

#### 4.2.3. Indian Pines

The Indian Pines hyperspectral dataset is a widely used benchmark in remote sensing for HSI classification and land-cover recognition. It was collected by the Airborne Visible/Infrared Imaging Spectrometer (AVIRIS; NASA Jet Propulsion Laboratory, Pasadena, CA, USA) over the Indian Pines region in Indiana, USA, in 1992. The image consists of 145 × 145 pixels, each associated with 200 spectral bands (out of the original 220, after removal of water-absorption bands) covering the 0.4–2.5 µm spectral range. The spatial resolution is 20 m, and the scene mainly contains agricultural fields and forested areas.

There are 16 land-cover classes with the following sample counts: alfalfa (46), corn-notill (1428), corn-mintill (830), corn (237), grass-pasture (483), grass-trees (730), grass-pasture-mowed (28), hay-windrowed (478), oats (20), soybean-notill (972), soybean-mintill (2455), soybean-clean (593), wheat (205), woods (1265), buildings-grass-trees-drives (386), and stone-steel towers (93).

The dataset contains 21,025 pixels in total, including 10,249 land-cover pixels (48.75%) and 10,776 background pixels (51.25%). The class distribution is highly skewed, with sample sizes ranging from 20 to 2455 pixels (a disparity of about 123×), and many agricultural classes—such as different types of corn and soybean—exhibit very similar spectral signatures. These characteristics make Indian Pines a challenging testbed that demands strong discriminative capability from HSI classification models.

The dataset is extensively used in hyperspectral image classification, land-cover recognition and segmentation, and in benchmarking machine learning and deep learning models. It is typically distributed in MAT file format with both spectral cubes and corresponding land-cover labels. Detailed data categories and sample counts are presented in Table 4. Data categories and number of samples. Since the annotations contain mixed and background pixels, background pixels are removed before analysis. The very high spectral dimensionality may cause a “curse of dimensionality”, so dimensionality reduction is routinely applied as a preprocessing step. The Indian Pines dataset has therefore become a key resource for hyperspectral remote sensing, providing rich experimental data for algorithm development and validation.

### 4.3. Evaluation Metrics

We evaluate hyperspectral image (HSI) classification performance using three core metrics: Average Accuracy (AA), Overall Accuracy (OA), and the Kappa Coefficient. These metrics jointly characterize model behavior on small-sample, high-dimensional data.

(14) $$AA=\frac{1}{n}\sum_{i=1}^{n}{recall}_{i}$$

AA measures per-class balance. It is defined as the arithmetic mean of class-wise recalls, as in (14), where denotes the recall of the class and n is the number of classes. A high AA indicates that the classifier performs consistently across all land-cover categories and is particularly informative when assessing SE(2)- and affine-Lie-group-based geometric features under class imbalance.

(15) $$OA=\frac{TP+TN}{TP+FN+FP+TN}$$

OA quantifies global performance by computing the proportion of correctly predicted samples over all test samples, as in (15). Using the confusion matrix, TP and TN denote correctly predicted positive and negative samples, while FP and FN denote the corresponding misclassified samples. OA thus provides a compact overall measure of pixel-level accuracy.

(16) $$\kappa =\frac{N\sum_{i=1}^{r}x_{ii}-\sum_{i=1}^{r}(x_{i+}\cdot x_{+i})}{N^{2}-\sum_{i=1}^{r}(x_{i+}\cdot x_{+i})}$$

The Kappa coefficient *κ* evaluates the agreement between predictions and ground truth after subtracting chance agreement. It offers a more stringent measure than OA, which is crucial for HSI tasks with high dimensionality and spectral redundancy, such as those explicitly targeted by the redundancy-elimination module (REM) in DGPF-RENet. Its formula, given in (16), involves the number of classes, the total number of evaluated pixels, the diagonal entries of the confusion matrix, and the row and column totals. Larger *κ* values indicate stronger consistency with the ground truth and hence better classifier reliability.

### 4.4. Comparative Experiments

To thoroughly examine the effectiveness and generalization of the proposed Lie-group-based geometric-invariant feature extraction model, we design a set of comprehensive comparative experiments. Our method is compared with nine representative HSI classification approaches that span classical machine learning, modern convolutional neural networks, and vision transformer architectures, as well as the strong HSI baseline DGPF-RENet [65]. Quantitative and qualitative results on multiple challenging datasets demonstrate that the proposed non-Euclidean geometric modeling confers clear advantages over conventional Euclidean-space methods when dealing with complex remote sensing scenes.

For clarity and consistency, all compared methods are now denoted using a unified naming scheme, namely Lee [66], Hybridformer [67] (Hybrid), SVM_grid [68] (SVM_g), ViT [1], Cnn2D [69], He [70], Sharma [71] (Shar), FDSSC [72], and DGPF, where DGPF refers to the DGPF-RENet baseline [65]. The same abbreviations are consistently adopted across the main text, tables, and figure captions, ensuring that each method is clearly identified and eliminating potential confusion caused by inconsistent naming in the original manuscript.

From a quantitative perspective, Table 5 reports the performance of all models on the WHU-Hi-HongHu dataset under a training protocol of 50 samples per class. “Params” refers to the number of trainable parameters for each model. Under this highly imbalanced setting, our method (Ours) achieves an overall accuracy (OA) of 95.77%, an average accuracy (AA) of 96.32%, and a Kappa coefficient of 94.67%, outperforming all competing approaches on all three metrics. Notably, despite a nearly 123-fold gap between the largest and smallest classes, the AA of 96.32% remains substantially higher than that of all competitors, including the strong baseline DGPF-RENet. This indicates that the model does not overfit majority classes, but instead maintains stable and balanced performance across all categories—a property directly attributable to the introduced explicit geometric constraints, which act as a powerful regularizer to prevent performance degradation on sparsely sampled classes. In order to further evaluate the robustness of our model under more stringent small-sample conditions, we performed additional experiments with 25 training samples per class for each category. Specifically, we used 10 repeated random hold-out splits. In each repeat, a new training subset was randomly sampled, and the samples used for training in one repeat were not reused as training samples in subsequent repeats, so that the 10 runs corresponded to distinct training realizations. We report mean ± SD over these repeated runs. The variation across the 10 runs remained small, while our method consistently maintained a clear advantage under this more challenging protocol.

For consistency with the official WHU-Hi-HongHu evaluation protocol, we compare 50-samples-per-class results only with methods reported under the same 50-samples-per-class setting, and 25-samples-per-class results only with methods reported under the corresponding 25-samples-per-class setting. Under this matched protocol, the published DGPF-RENet results reported by Zhan [65] are 95.76% OA for 50 samples per class and 91.33% OA for 25 samples per class, while EMNet achieves 95.77% and 90.98%, respectively. Although EMNet is slightly lower in the 25-samples-per-class case, the gap is very small, indicating that the proposed method remains on par with this strong recent approach while offering a distinct geometry-aware modeling perspective.

To quantify the reliability of the reported means, we report mean ± SD and compute the standard error of the mean as SE = SD/sqrt(*n*) (*n* = 10). On the three benchmark datasets, the absolute gains of EMNet over the strongest baseline DGPF-RENet are clearly larger than the corresponding sampling uncertainty: on WHU-Hi-HongHu (50 samples/class), OA improves from 92.43 ± 0.42% to 95.77 ± 0.19% (+3.34 pp); on Houston2013 (50 samples/class), from 95.26 ± 0.21% to 97.37 ± 0.14% (+2.11 pp); and on Indian Pines (5% labels), from 94.37 ± 0.11% to 96.09 ± 0.11% (+1.72 pp). The 95% confidence intervals of the two methods are well separated in these settings, and a conservative Welch t-test on the 10-run results indicates statistically significant improvements (*p* < 1 × 10^−8^) for OA/AA/Kappa. In addition, EMNet improves class-wise accuracy for 21/22 classes on WHU-Hi-HongHu, 13/15 on Houston2013, and 13/16 on Indian Pines, confirming that the gain is not driven by a small subset of categories. Following common reporting practice, all values are rounded to match the precision justified by the corresponding run-to-run variation.

Table 6 summarizes the comparative results on the Houston2013 dataset, again using 50 labeled samples per class for training. In this setting, dominated by regular man-made structures, the advantage of the proposed model is further amplified, achieving 97.37% OA, 97.74% AA, and a Kappa of 97.16%. All key metrics rank first, revealing a clearly dominant performance. These results strongly confirm the central role of explicit geometric modeling in structured urban scenes. Owing to frequent viewpoint changes, urban objects exhibit complex geometric transformations, whereas the implicit geometric modeling in the baseline—essentially limited to approximate scale handling—struggles in such scenarios. In contrast, by jointly exploiting the SE(2) group and the affine Lie group, our network can faithfully encode translations, rotations, and more general affine deformations, tightly coupling the model architecture with the intrinsic geometric structure of the data.

Finally, Table 7 presents the comparison on the Indian Pines dataset, where 5% of all labeled pixels are used for training. Our model again demonstrates both strong performance and good generalization, obtaining 96.09% OA, 94.75% AA, and a Kappa of 95.54%, consistently surpassing all competitors. The main challenge of this dataset lies in discriminating crop categories with extremely similar spectral signatures, which places stringent demands on the model’s fine-grained discriminative capacity. The success of our approach shows that the proposed geometric framework effectively mitigates spectral ambiguity by enforcing spatial geometric consistency. The enforced geometric equivariance drives the network to move beyond isolated spectral responses and instead learn local structural and textural context, providing strong spatial regularization that markedly enhances the separability of spectrally confounded classes.

When we move from these quantitative tables to the classification maps, the performance differences become even more visually apparent. The numerical gains are corroborated by cleaner and more coherent spatial distributions, which clearly expose the fundamental gaps among different methods in terms of structure preservation and noise suppression.

**Figure 2:**
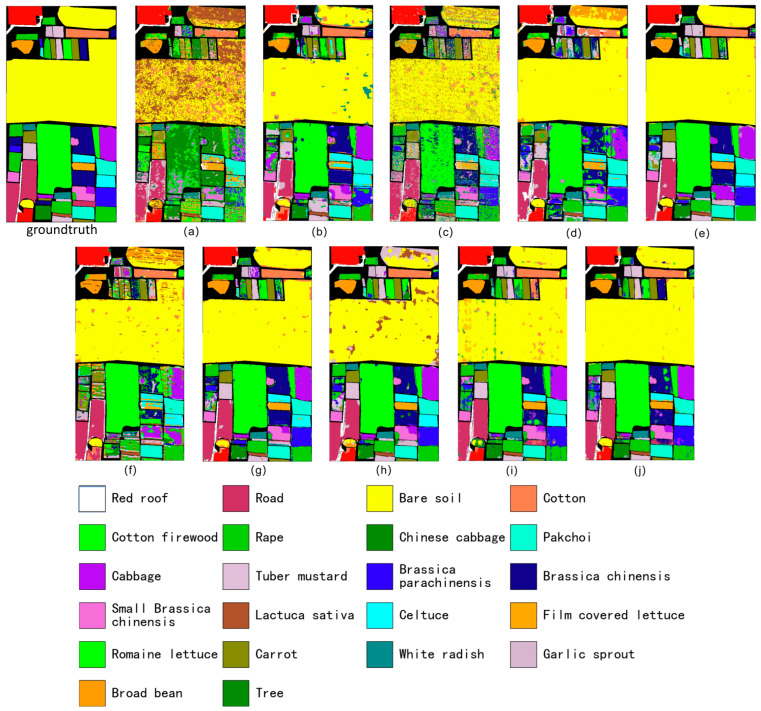
Classification visible results for the WHU-Hi-HongHu HSI by (**a**) Lee, (**b**) Hybridformer, (**c**) SVM_grid, (**d**) Vit, (**e**) Cnn2D, (**f**) He, (**g**) Sharma, (**h**) FDSSC, (**i**) DGPF-RENet, and (**j**) Ours.

As shown in Figure 2, the classification maps on the WHU-Hi-HongHu dataset clearly highlight the advantages of the proposed method. The result produced by our model (Figure 2j) exhibits markedly improved smoothness and spatial consistency compared with all competitors. Large cropland regions are almost perfectly homogeneous, with virtually no mislabeled pixels, forming coherent parcels that closely follow the true object boundaries. In contrast, the other methods (Figure 2a–h) suffer from pronounced “salt-and-pepper” noise, fragmented and blurry boundaries, and heavy class mixing. Even relative to the strong baseline DGPF-RENet (Figure 2i), our method shows superior robustness in regions where classes are intertwined, producing sharper and cleaner delineations along object edges.

Beyond overall smoothness, Figure 2 reveals a more practical point that is easy to miss in purely numerical tables: most errors are not evenly distributed, but concentrate as small, isolated “salt” pixels inside otherwise homogeneous parcels. A representative case is the large bare-soil region in the upper-middle part of the scene (yellow). Several baselines still leave many scattered vegetated labels within this parcel—typically green- or orange-toned crop classes—which means bare soil is spuriously interpreted as crops. In Figure 2i, these false alarms appear as sparse dots and short streaks crossing the bare-soil area, indicating that the model remains sensitive to local spectral fluctuations and mixed pixels even when the global OA is high. Our result in Figure 2j largely removes these artifacts: the bare-soil parcel becomes almost uniformly yellow, and the few residual outliers are strongly reduced. This change is not cosmetic. In agricultural mapping, a handful of isolated crop pixels inside a bare-soil field can translate into wrong acreage estimates or false detections. The improvement suggests that the proposed SE(2)-equivariance stabilizes the response under pose variations, while the affine-aware CFC suppresses redundant and deformation-induced activations, so that label decisions are driven more by parcel-level structure than by pixel-level noise. A second typical failure mode is boundary leakage along narrow field strips near the top edge of the scene: competitors often blur the boundaries between adjacent crops, whereas Figure 2j follows the long, thin parcel shapes more faithfully and keeps edges crisp.

**Figure 3:**
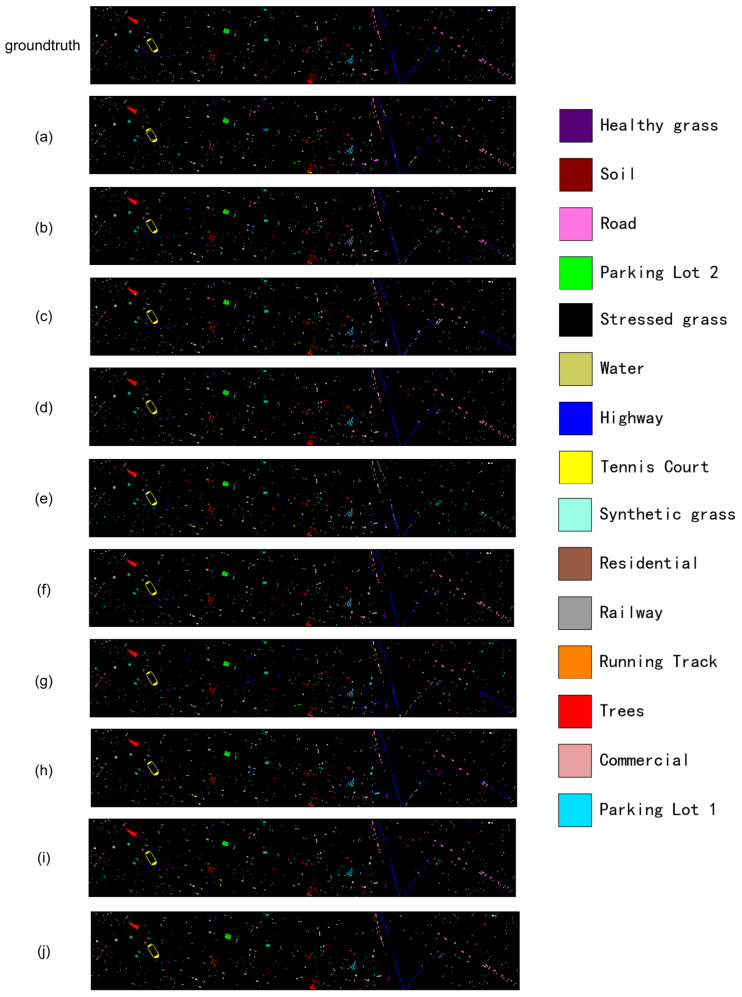
Classification maps for the Houston2013 HSI produced by (**a**) Lee, (**b**) Hybridformer, (**c**) SVM_grid, (**d**) ViT, (**e**) CNN2D, (**f**) He, (**g**) Sharma, (**h**) FDSSC, (**i**) DGPF-RENet, and (**j**) Ours.

A similar observation can be made on the Houston2013 dataset in Figure 3, which again highlights the structural preservation capability of our model for man-made objects. In Figure 3j, roads and highways are continuously and accurately traced, while parking lots and buildings exhibit clear outlines and noise-free interiors with intact geometric shapes. Most competing methods, by contrast, fail to preserve such geometric structures: roads appear broken, and large commercial or residential areas are heavily contaminated by misclassified pixels from other categories. These visual patterns offer direct evidence that explicit geometric modeling enables our network to better capture and maintain structured information in urban scenes.

Figure 3 provides an urban counterpart where the “cost” of small errors is even more explicit, because many categories correspond to thin, man-made structures. The right half of the scene contains a prominent highway corridor (blue) together with nearby road segments (pink) and rail-like structures (gray). For several competing methods, these linear objects become intermittently broken, and the missing segments are filled by visually plausible but incorrect labels (e.g., highway pixels turning into road or railway, or vice versa). Such discontinuities are particularly problematic in remote-sensing cartography, where connectivity and topology matter as much as per-pixel accuracy. In contrast, our prediction in Figure 3j keeps these lines more continuous and better aligned with the underlying geometry, with noticeably less label bleeding around the edges. This is also visible on compact regions such as parking lots (cyan/green) and built-up blocks (brown/peach): many baselines show pepper noise inside these regions, while our map yields cleaner interiors and sharper outlines, which is closer to how these objects appear in real maps. Notably, the small tennis-court area highlighted on the left (yellow) is preserved with an intact rectangular shape in Figure 3j, whereas other results tend to fragment it or contaminate it with neighboring categories. These qualitative differences support our claim that explicit geometric constraints do not merely improve average accuracy but reduce application-critical local mistakes.

**Figure 4:**
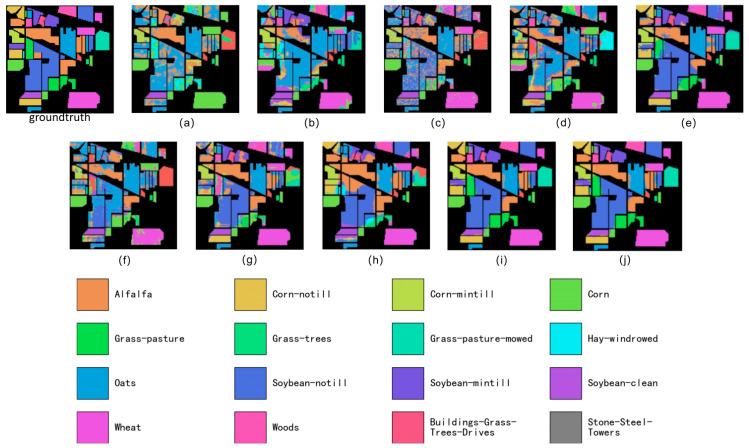
Classification maps for the Indian Pines HSI produced by (**a**) Lee, (**b**) Hybridformer, (**c**) SVM_grid, (**d**) ViT, (**e**) CNN2D, (**f**) He, (**g**) Sharma, (**h**) FDSSC, (**i**) DGPF-RENet, and (**j**) Ours.

Figure 4 reports the results on the classical Indian Pines dataset, which places particularly stringent demands on the use of spatial information. The map generated by our model (Figure 4j) contains large, homogeneous patches that closely match the actual layout of the fields; parcel boundaries are sharp and easily distinguishable, effectively addressing the “same object, different spectra” and “different objects, similar spectra” phenomena. This indicates that the model successfully leverages spatial context to disambiguate spectrally similar classes. In contrast, most competing methods produce mottled and fragmented maps, a typical consequence of relying predominantly on pixel-wise spectral signatures while neglecting spatial structure. The superior visual quality of our results provides strong qualitative evidence that the proposed geometric invariance effectively suppresses spectral confusion.

On Indian Pines, the qualitative comparison is especially informative because the dataset contains many agricultural classes with extremely similar spectra. Here, the main difficulty is not recognizing isolated objects, but keeping each field internally consistent and separating adjacent fields with subtle appearance changes. In Figure 4, several baselines still exhibit a “mottled” texture inside large parcels, most noticeably within the dominant soybean and corn fields, where scattered pixels flip to spectrally close categories. For instance, in the large blue soybean parcel on the left side of the scene, competing maps include visible speckles of corn- or grass-like labels, suggesting that local spectral variation overrides spatial context. Our output in Figure 4j markedly reduces this intra-field salt-and-pepper effect, producing more uniform regions that respect the field boundaries. The benefit becomes clearer around the mid-right area that contains narrow, band-shaped fields: thin parcels are easily broken or merged by other methods, but Figure 4j preserves their elongated geometry and keeps the class transitions concentrated at the true borders rather than scattered inside the fields. In practical crop mapping, these are exactly the errors that are least tolerated—small islands of a wrong crop type inside a field can trigger false agronomic decisions—so the improved spatial coherence in Figure 4j provides an important qualitative complement to the OA/AA/Kappa gains.

Taken together, the rigorous quantitative results in Table 3, Table 4 and Table 5 and the intuitive qualitative comparisons in Figure 2, Figure 3 and Figure 4 consistently demonstrate the superiority of the proposed model. The performance gains do not stem from deeper architectures or aggressive parameter tuning, but from a fundamental advance in the underlying theory. By explicitly integrating SE(2) and affine Lie-group structures into the network, we address intrinsic limitations of prior approaches at the mathematical level. Unlike the baseline, whose PFM module only implicitly and incompletely approximates scale invariance, our method explicitly and comprehensively models translations, rotations, scaling, and shear transformations. Consequently, the model is no longer constrained by Euclidean assumptions and can operate directly on the intrinsic geometric consistency of the data. This deeper mathematical formulation enables precise and robust exploitation of the joint spatial–spectral geometry of hyperspectral data, yielding state-of-the-art performance under the canonical challenges of small sample size, high dimensionality, and strong class confusion.

### 4.5. Experiment on Large-Scale Urban Scene Data

To further examine whether the proposed geometry-guided design can generalize beyond the three commonly used benchmarks (WHU-Hi-HongHu, Houston2013, and Indian Pines), we additionally evaluate EMNet on two public hyperspectral datasets that exhibit stronger distribution shift and more severe long-tail class imbalance. The three standard benchmarks are widely accepted and remain non-trivial under strict few-shot budgets; however, their overall accuracies for modern models are already high, meaning that further improvements often come from correcting a relatively small fraction of residual errors. Even under these mature settings, EMNet still achieves clear gains, indicating a consistent reduction in residual mistakes. A complementary evaluation on harder scenes is therefore helpful for judging robustness in settings where nuisance variation and imbalance are more pronounced.

#### 4.5.1. Motivation and Newly Introduced Datasets

The hyperspectral community typically validates new methods on a small set of well-curated datasets. This practice ensures fair comparison, but it can understate the value of inductive biases once performance approaches saturation on long-studied benchmarks. To stress-test generalization, we introduce OHID-1 [73] and Xiongan [74], which differ markedly from the canonical benchmarks in sensing conditions, scene composition, and class distributions. Both datasets present challenging mixtures of urban textures, background dominance, and minority classes, which are known to amplify sampling sensitivity in few-shot protocols.

Xiongan is a large-scale hyperspectral scene of 1580 × 3750 pixels with 256 spectral bands. The dataset provides pixel-wise labels for 21 categories and totals 5,925,000 labeled pixels. It exhibits an extremely long-tail distribution: the background category alone accounts for 2,247,890 pixels, while several minority categories contain fewer than 5000 pixels. Such an imbalance, together with complex land-cover mixtures and strong intra-class variability, makes Xiongan a representative stress test for few-shot generalization.

OHID-1 is constructed from Orbita hyperspectral data and depicts diverse urban and peri-urban areas. It contains 10 labeled hyperspectral images with 32 spectral bands and a spatial size of 512 × 512 pixels. Seven land-cover categories are included (Building, Farmland, Forest, Road, Water, Bareland, and Fishpond). The class distribution is imbalanced, ranging from 661,721 pixels in the largest class to 14,699 pixels in the smallest class.

The detailed class counts of both datasets are summarized in Table 8, which highlights the pronounced imbalance that can exacerbate confusion among visually similar categories and increase run-to-run variability under limited supervision.

#### 4.5.2. Experimental Protocol

We follow the same training configuration as in the main experiments to keep the evaluation controlled and comparable. For each dataset, we report results under two training budgets: (i) 50 labeled samples per class and (ii) 1% of labeled samples per class. For each budget, we use 10 repeated random hold-out splits rather than a 10-fold partition. In each repeat, the training set is randomly sampled from the labeled pixels, and the samples selected for training in one repeat are not reused as training samples in subsequent repeats, so that the 10 training subsets are distinct. All remaining labeled pixels in that repeat are used for testing. We report the mean performance together with the standard deviation (SD) across the 10 runs, and compute the standard error as SE = SD/sqrt(10) to indicate the precision of the estimated mean. Unless otherwise stated, all implementation details remain identical to those used in the benchmark experiments. Importantly, all performance comparisons in this section are conducted within the same training budget, and we avoid cross-budget (cross-sample-number) comparisons.

#### 4.5.3. Experimental Results and Discussion

On OHID-1 under the 1% protocol, EMNet improves OA from 84.60 ± 0.11% to 93.59 ± 0.08%, AA from 86.38 ± 0.13% to 91.09 ± 0.09%, and Kappa from 78.47 ± 0.15% to 91.72 ± 0.11% (Table 7). Beyond the mean gains, the reduction in dispersion is also clear: the OA variability decreases (SD 0.11 → 0.08), suggesting that the proposed geometric constraints contribute to a more stable decision boundary under random sampling.

On the more challenging Xiongan dataset under the same 1% protocol, EMNet raises OA from 85.89 ± 0.11% to 93.77 ± 0.08%, increases AA from 93.55 ± 0.16% to 97.31 ± 0.03%, and boosts Kappa from 83.73 ± 0.13% to 92.68 ± 0.09% (Table 8). Meanwhile, the OA dispersion decreases (SD 0.11 → 0.08), indicating that EMNet not only improves the mean accuracy but also reduces the likelihood of unfavorable random splits under long-tail imbalance.

Taken together, these results support a consistent interpretation: when evaluation is performed on mature benchmarks with already high accuracies, the observable margin is naturally compressed, and improvements may appear incremental. Nevertheless, EMNet still delivers stable gains under those near-ceiling conditions in the main experiments, which implies that the proposed explicit geometric modeling can correct residual errors that remain difficult for purely Euclidean designs. When the datasets become harder—due to stronger distribution shift, heavier imbalance, and more complex scene mixtures—the advantage of the geometry-guided inductive bias becomes more pronounced, yielding clearer improvements in both accuracy and stability. Importantly, these gains are achieved with only a small increase in parameters, as summarized in Table 7, which preserves the practical efficiency profile of the baseline.

#### 4.5.4. Per-Class Results

To better understand where the improvements arise, we further report per-class accuracies for both datasets.

For OHID-1, the per-class results under the 1% protocol are listed in Table 9. EMNet yields notable gains on several confusing categories, including Class 1 (77.74% → 94.34%), Class 3 (88.87% → 96.30%), Class 4 (65.92% → 73.90%), and Class 5 (83.79% → 95.10%). Meanwhile, a few classes remain comparable or fluctuate slightly (e.g., Class 2: 95.99% → 94.00%; Class 6: 100.00% → 90.21%), suggesting that the overall improvement is mainly driven by better separation on the harder categories rather than uniformly increasing all classes.

For Xiongan, the per-class results under the 1% protocol are summarized in Table 10. While many dominant categories are already close to saturation for both methods, EMNet still improves several harder classes, such as Class 16 (76.97% → 91.52%), Class 14 (84.58% → 95.47%), Class 13 (85.40% → 94.62%), and Class 19 (58.98% → 64.17%). This class-wise behavior is consistent with the intended role of explicit geometric constraints: rather than relying on heavier parameterization, EMNet regularizes the transformation behavior of intermediate features and stabilizes learning on the harder, more ambiguous categories under scarce and imbalanced supervision.

In summary, these dataset generalization experiments provide complementary evidence beyond the standard benchmarks. While performance on long-studied datasets is already relatively high, EMNet still delivers repeatable gains. More importantly, on challenging scenes such as OHID-1 and Xiongan, where distribution shift and severe class imbalance make the task substantially harder, EMNet achieves large and stable improvements across random splits. This pattern indicates that the proposed geometry-guided design is not exploiting chance but contributes a robust inductive bias that improves reliability under more demanding conditions, thereby supporting the effectiveness and robustness of EMNet.

### 4.6. Statistical Analysis

To complement the quantitative comparisons in Table 3, Table 4, Table 5, Table 6, Table 7 and Table 8, we provide statistical evidence from three perspectives. First, we use Yule’s Q statistic to quantify the similarity of class-wise performance patterns between EMNet and each baseline. Second, we apply the Friedman test followed by the Nemenyi post hoc procedure to examine whether the overall performance rankings are statistically significant across multiple benchmark settings. Third, we report confidence intervals and effect sizes under the cross-dataset generalization protocol in Table 7 and Table 8 to describe uncertainty and practical relevance.

#### 4.6.1. Q-Statistic-Based Difference Analysis

Motivated by the Q-statistic analysis in Table 8 and Table 9 of the reference paper, we adopt Yule’s Q statistic to measure the agreement between EMNet and a competing method in terms of class-wise performance tendencies. For each dataset, we discretize per-class accuracies into a binary indicator that reflects whether a class is relatively well recognized by a method.

(17) $$Q=\frac{N_{11}N_{00}-N_{10}N_{01}}{N_{11}N_{00}+N_{10}N_{01}}$$

We avoid using a fixed global threshold because such a threshold can be biased by dataset difficulty, and instead apply a method-wise median criterion. For a method m, class c is marked as well recognized when Acc_m(c) exceeds the median per-class accuracy of m on that dataset. Using the contingency counts N11, N00, N10, and N01, the Q statistic is computed in Equation (17). Here, N11 denotes the number of classes that are well recognized by both EMNet and the compared method, N00 the number of classes that are well recognized by neither method, and N10/N01 the numbers of classes on which only one of the two methods performs above its own median. The value of Q lies in [−1, 1]: Q→1 indicates highly similar class-wise behavior, Q≈0 indicates weak association, and Q < 0 suggests complementary strengths across classes.

As summarized in Table 11, EMNet achieves the best averaged OA, AA, and Kappa across the evaluated benchmarks, and DGPF is the closest competitor in terms of absolute accuracy.

Table 12 shows that DGPF yields the highest Q value among all baselines, and its weighted average Q is about 0.901. This indicates that DGPF is the most behaviorally similar method to EMNet in terms of which classes are relatively easy or hard. In contrast, methods with clearly lower averaged accuracies tend to exhibit smaller Q values, revealing larger mismatches in class-wise strengths and weaknesses compared with EMNet.

#### 4.6.2. Friedman Test and Nemenyi Post Hoc Analysis

To further verify whether the overall performance differences among multiple methods are statistically significant across different benchmark settings, we adopt the Friedman test, which is a non-parametric alternative to repeated-measures ANOVA based on per-setting ranks. Let r_{i,j} denote the rank of method j on evaluation setting i, where a smaller rank indicates better performance, and let R_j = Σ_i r_{i,j} be the rank sum of method j over N settings among k methods.

(18) $$\chi_{F}^{2}=\frac{12N}{k(k+1)}\sum_{j=1}^{k}R_{j}^{2}-3N(k+1)$$

The Friedman statistic is given in Equation (18), and we additionally report the Iman–Davenport correction in Equation (19) for a less conservative assessment.

(19) $$F_{F}=\frac{(N-1)\chi_{F}^{2}}{N(k-1)-\chi_{F}^{2}}$$

Equation (19) provides an F-distributed approximation that improves the test power under a small number of evaluation settings.

(20) $$CD=q_{\alpha }\sqrt{\frac{k(k+1)}{6N}}$$

For post hoc pairwise comparisons, we use the Nemenyi procedure and compute the critical difference as shown in Equation (20). The parameter q_α is obtained from the studentized range distribution and controls the family-wise error rate.

In this study, we conduct the test on OA over four evaluation settings: WHU-Hi-HongHu with 50 samples per class, WHU-Hi-HongHu with 25 samples per class, Houston2013 with 50 samples per class, and Indian Pines with 5% training samples. The Friedman test yields χF^2^ = 30.05 with p = 4.29 × 10^−4^, and the Iman–Davenport statistic yields FF = 15.17 with p = 2.04 × 10^−8^, indicating that the performance differences among the compared methods are statistically significant.

Table 13 lists the resulting average ranks, where smaller values indicate better performance. With α = 0.05 and k = 10 methods, the Nemenyi critical difference is CD = 6.77. Given that we have only four benchmark settings, this post hoc test is intentionally conservative and may fail to separate strong methods with close ranks. Therefore, in addition to the rank-based analysis, we also conduct direct split-wise comparisons between EMNet and the strongest baseline DGPF-RENet on each dataset over 10 random splits. These per-dataset comparisons consistently support statistically significant improvements for OA/AA/Kappa (p < 1 × 10^−8^ under a conservative Welch test), providing complementary evidence beyond the multi-method ranking test.

#### 4.6.3. Confidence Interval and Effect Size Analysis

Besides the rank-based significance analysis, we quantify the uncertainty of the reported mean OA in the cross-dataset generalization experiments in Table 7 and Table 8, which are obtained over 10 random splits.

(21) $$CI_{95\%}=\bar{x}\pm t_{n-1,0.975}\cdot \frac{s}{\sqrt{n}}$$

We compute the 95% confidence interval of the mean using Equation (21), where $\bar{x}$ and s denote the sample mean and standard deviation, and t_{n − 1,0.975} is the critical value of the t distribution.

(22) $$d=\frac{{\bar{x}}_{1}-{\bar{x}}_{2}}{s_{p}},\;\;\;\;\;\;s_{p}=\sqrt{\frac{(n_{1}-1)s_{1}^{2}+(n_{2}-1)s_{2}^{2}}{n_{1}+n_{2}-2}}$$

To reflect practical significance, we further report Cohen’s d as a standardized effect size in Equation (22), where s_p is the pooled standard deviation of the two methods.

As shown in Table 14, EMNet provides consistent and substantial OA gains on both OHID-1 and Xiongan under different training budgets. The 95% confidence intervals of EMNet and DGPF do not overlap in any case, indicating that the improvement is stable across random splits. The effect sizes measured by Cohen’s d are extremely large, which supports that the observed gains are not only statistically reliable but also practically meaningful.

### 4.7. Ablation Studies

To rigorously quantify the contribution of each proposed geometric component, we conduct ablation studies on the WHU-Hi-HongHu dataset. Starting from the unified baseline DGPF-RENet, we progressively remove, insert, or replace modules while keeping the remaining settings fixed. This protocol isolates the effects of the SE(2)-based equivariance guidance module and the affine Lie-group-based characteristic filtering convolution (CFC), and it also allows us to evaluate whether the two modules provide complementary (synergistic) gains.

#### 4.7.1. Overall Ablation

We begin with overall ablations that measure the net gain obtained by adding the two geometric modules to the baseline.

Table 15 summarizes the results. As a strong starting point, DGPF-RENet reaches 92.43% OA, 93.04% AA, and 90.53% Kappa on WHU-Hi-HongHu under the adopted protocol.

Adding the SE(2)-based equivariance guidance module alone (DGPF-RENet + EGM) produces consistent improvements, raising OA/AA/Kappa to 92.71%/93.14%/90.86%. This indicates that explicitly enforcing equivariance to rigid motions (translation and rotation) enhances robustness to pose variation and compensates for the baseline’s lack of dedicated mechanisms beyond approximate scale handling.

By contrast, introducing only the CFC (DGPF-RENet + CFC) changes OA/AA/Kappa by at most 0.02–0.03 percentage points relative to the baseline (Table 13), i.e., essentially no net gain. This indicates that CFC is not intended to be used as an isolated plug-in; without first stabilizing features against basic rigid transformations, directly modeling more complex affine deformations is difficult to exploit reliably during optimization. Once EGM provides a stable SE(2)-equivariant backbone, CFC becomes effective and yields the large boost observed in the full model.

Once the two modules are combined (DGPF-RENet + EGM + CFC), performance increases markedly, achieving 95.77% OA, 96.32% AA, and 94.67% Kappa. The gain demonstrates a clear synergy: the SE(2)-based module provides a stable geometric backbone for feature extraction, after which the affine Lie-group CFC can more effectively capture and correct scaling/shearing-induced feature drift while filtering redundant responses. Together, they implement a hierarchical geometric-invariance strategy that delivers the final performance boost.

#### 4.7.2. Effectiveness of the Equivariant Guidance Module

To better understand how the equivariance guidance module works internally, we perform component-level ablations for this module (denoted as EMG in the ablation labels). EMG consists of (1) an SE(2) transform layer that explicitly warps features via learnable rotation/translation parameters, and (2) an attention-based graph-guided fusion block (AttnFuse) that aggregates and compresses the geometric and semantic streams.

Using the CFC-only setting as the reference, we remove these components individually or jointly and report the results in Table 16. Relative to the CFC baseline, the full EMG (CFC + EMG) brings a +3.36 percentage-point OA gain (92.41% → 95.77%), confirming the effectiveness of explicit equivariance guidance. Removing the SE(2) transform (EMG_noTransform) reduces OA to 92.82%, i.e., a 2.95-point drop compared with the full EMG, which highlights the non-negligible benefit of explicitly modeling translation/rotation equivariance.

Removing AttnFuse has an even larger impact: replacing it with standard convolutional compression (EMG_noAttnFuse) lowers OA to 92.63%, a 3.14-point decrease from the full EMG. When both components are removed (EMG_noAttnFuse_noTransform), the OA (92.64%) is almost identical to EMG_noAttnFuse (92.63%), implying that the geometric information introduced by SE(2) transforms cannot be fully utilized without an effective attention-driven fusion pathway. In other words, SE(2) transformation and attention-guided fusion must operate together to unlock the module’s full potential.

**Figure 5:**
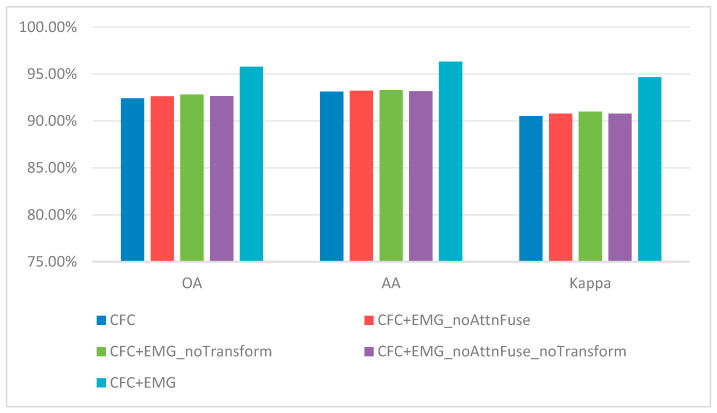
Ablation study of the EMG.

Figure 5 visualizes these results. The full configuration (CFC + EMG) consistently achieves the best OA, AA, and Kappa, whereas ablating either the SE(2) transform or AttnFuse leads to clear degradation, further supporting their complementary roles.

In summary, the EMG ablation results underscore that performance gains stem from the coordinated design rather than a single component. Neither SE(2) transforms alone, nor is attention fusion alone sufficient to reach the best accuracy. The sharp drop observed when removing AttnFuse suggests that equivariant features must be aggregated through an expressive, attention-based fusion mechanism so that subsequent layers can exploit the geometric stability while preserving semantic discrimination.

#### 4.7.3. Effectiveness of the Characteristic Filtering Convolution

**Figure 6:**
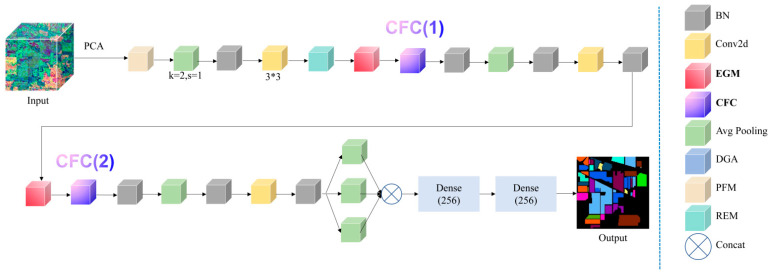
Integration of CFC(1) and CFC(2) in the proposed network. The legend colors denote BN (gray), Conv2d (yellow), EGM (red), CFC (purple), Avg Pooling (green), DGA (blue), PFM (beige), and REM (cyan); the circle denotes concat.

After verifying the overall effectiveness, we further analyze the characteristic filtering convolution (CFC) by studying where it should be inserted. These ablations are performed on a stable backbone where EMG has already been included. As illustrated in Figure 6, we replace two original Conv1d layers in the spectral branch with CFCs in different ways: replacing only the first Conv1d (CFC(1)), replacing only the second Conv1d (CFC(2)), or replacing both (CFC(1) + (2)).

The corresponding results are reported in Table 17. Among all variants, EMG + CFC(2) achieves the best performance (95.77% OA, 96.32% AA, 94.67% Kappa), matching the full model. In comparison, EMG + CFC(1) yields only a modest improvement (OA 92.92%), indicating that applying affine Lie-group filtering at deeper stages—where features are more abstract and semantically organized—is more beneficial than applying it to shallow, low-level representations.

Interestingly, stacking both CFC(1) and CFC(2) (EMG + CFC(1) + (2)) leads to a noticeable drop (OA 92.83%). This observation suggests that simply adding more geometric constraints does not guarantee better performance; redundant or misaligned invariance priors can introduce optimization conflicts or unnecessary complexity, ultimately hurting generalization. Therefore, CFC(2) represents the most appropriate insertion point for balancing geometric modeling capacity and learning stability.

**Figure 7:**
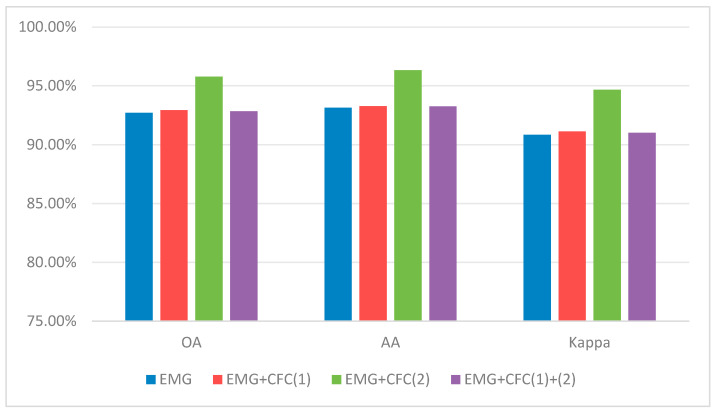
Ablation results for the CFC placement.

Figure 7 provides a visual summary. Inserting CFC(2) at the second Conv1d consistently yields the best OA/AA/Kappa and clearly outperforms CFC(1), while the combined setting (CFC(1) + (2)) underperforms the single CFC(2) insertion, reinforcing that “more modules” is not equivalent to “better modules” in this context.

Overall, these ablations clarify the role and placement of the affine Lie-group module: its effectiveness is strongly position-dependent, and the greatest gains arise when it operates on deeper, more abstract features. Moreover, the degradation observed for CFC(1) + (2) suggests a practical design guideline—complex geometric modules should be integrated minimally and precisely, avoiding redundant constraints. These findings justify our choice of CFC(2) and provide guidance for embedding Lie-group operators into deep HSI networks.

## 5. Additional Robustness Analysis and Conclusions

### 5.1. Preliminary Experiment Under Synthetic Rain and Snow Perturbations

To provide a more concrete estimate of the reviewer’s concern, we further conducted a preliminary experiment on Indian Pines under the same 5% labeled-sample protocol used in the main study. Specifically, we constructed synthetic rain and synthetic snow versions through image-level perturbation to simulate adverse weather-induced appearance degradation, and then re-evaluated both the strongest baseline (DGPF-RENet) and EMNet. Although these perturbations cannot fully reproduce real rainfall, snowfall, cloud shadow, surface wetness, or cross-season phenological variation in hyperspectral observations, they provide a controlled first-step estimate of weather-related sensitivity.

As shown in Table 18, EMNet consistently degrades much less than DGPF-RENet under both perturbations. From the clean condition to synthetic rain, the OA/AA/Kappa drops of EMNet are 1.19/3.92/1.35 percentage points, whereas the corresponding drops of DGPF-RENet are 5.91/11.90/6.70 percentage points. Under synthetic snow, the drops of EMNet are 2.36/6.02/2.68 percentage points, while DGPF-RENet decreases by 11.59/21.10/13.17 percentage points. These preliminary results do not replace real multi-temporal evaluation, but they do provide an initial quantitative estimate showing that the proposed geometry-guided design remains markedly more robust than the baseline under severe weather-like corruption.

### 5.2. Conclusions

To conclude, we focus on small-sample hyperspectral image classification under high labeling cost and strong geometric disturbances, and develop an explicit geometric-invariance framework that deeply couples Lie group theory with joint spectral–spatial modeling. The first component, an SE(2)-based module, encodes equivariance to translations and rotations, thereby strengthening pose robustness. On WHU-Hi-HongHu, augmenting DGPF-RENet with EMG improves OA from 92.43% to 92.71%, AA from 93.04% to 93.14%, and Kappa from 90.53% to 90.86%, i.e., gains of approximately 0.28, 0.10, and 0.33 percentage points. The second component, an affine Lie group–based manifold module, leverages this SE(2) backbone to better accommodate scale, shear, and other complex deformations: on WHU-Hi-HongHu, upgrading from EMG-only to the full EMG+CFC configuration boosts OA/AA/Kappa from 92.71%/93.14%/90.86% to 95.77%/96.32%/94.67%, with increments of about 3.06, 3.18, and 3.81 percentage points. On Houston2013 and Indian Pines, the complete model also consistently surpasses DGPF-RENet, raising OA from 95.26% and 94.37% to 97.37% and 96.09%, AA from 96.01% and 88.74% to 97.74% and 94.75%, and Kappa from 94.87% and 93.58% to 97.16% and 95.54%. Beyond the main experimental settings, we further evaluate a 25-sample-per-class protocol (10 random splits). EMNet achieves 90.98 ± 0.32% OA, 91.48 ± 0.17% AA, and 88.74 ± 0.38% Kappa, outperforming the DGPF-RENet baseline (87.24 ± 0.48% OA, 87.79 ± 0.23% AA, 84.18 ± 0.56% Kappa) under more severe label scarcity. In addition, large-scale evaluations on OHID-1 and Xiongan New Area, the proposed geometry-guided design yields clear accuracy and stability gains; under the 1% labeled-sample protocol, EMNet yields clear accuracy and stability gains on both large-scale datasets: on OHID-1, OA improves from 84.60% to 93.59%, and on Xiongan, OA improves from 85.89% to 93.77%, supporting its suitability for city-scale, long-tail hyperspectral mapping. From a mathematical standpoint, our approach moves beyond the standard Euclidean assumption and establishes an interpretable geometric-invariance modeling paradigm for HSI, substantially improving small-sample classification performance while pushing forward the theoretical and algorithmic boundary of small-sample hyperspectral learning. In addition, statistical analyses further corroborate the reported gains: the Friedman test on OA across four evaluation settings rejects the null hypothesis (χF^2^ = 30.05, p = 4.29 × 10^−4^), and the Iman–Davenport correction also indicates strong significance (FF = 15.17, p = 2.04 × 10^−8^). Moreover, the strongest baseline DGPF shows the highest behavior similarity to EMNet (weighted Q ≈ 0.901), while the 95% confidence intervals and Cohen’s d effect sizes under repeated splits confirm that the improvements are not only statistically significant but also practically meaningful. Future work will extend this preliminary perturbation-based evidence to real rainy, snowy, cloud-shadowed, and multi-temporal/cross-season hyperspectral datasets, and will also investigate deep multimodal fusion of HSI with RGB, LiDAR, SAR, and related sensors, combining Lie group geometry with cross-modal alignment and joint representations to further enhance generalization in complex and cross-domain environments.

## Figures and Tables

**Table 1:** Detailed specification of the newly introduced modules in Figure 1.

Section	Layer	Setting	Section	Layer	Setting
EGM branch	SE2Transform	theta + shift	CFC branch	LieGroupConv1D	k = 3, s = 1
	SE2 input	H × W × C		Filters	64
	AttmFuse	1 × 1, sigmoid		Affine params	scale + shift
	AttmFuse out	H × W × 1		Padding	same
	SE2GraphFusion	H × W × 2C		Position	spectral Conv1D-2
	EGM output	H × W × 64		Input patch	19 × 19 × num_PC

**Table 2:** Hardware and software configuration.

Hardware Environment		Software Environment	
CPU	Intel(R) Xeon(R) Platinum 8255C CPU @ 2.50 GHz	OS	Linux
RAM	40 GB	CUDA	9.2
Video memory	11 GB	Python	3.6.5
GPU	NVIDIA GeForce RTX 2080 Ti	Tensorflow	1.10.0
Server platform	autodl	Keras	2.2.0

**Table 3:** Summary of training method, network settings, spatial window size, and other hyperparameters.

Section		Parameter Setting	Section		Parameter Setting
Training method	Optimizer	RMSprop	Network settings	Fusion filters	96
	Loss function	Categorical cross-entropy		RE-attention stages	2
	Initial learning rate	1 × 10^−3^		First spectral layer	Conv1D
	Decay strategy	Exponential decay		Classification head	Dense 256, Dropout 0.5, Softmax
	Batch size	64	Spatial window size	Local patch size	19 × 19
	Training epochs	64		Input tensor shape	19 × 19 × num_PC
Network settings	Backbone type	demo backbone	Other hyperparameters	Repeated runs	10
	PFM branches	6		Train-test split	Non-disjoint
	Filters per PFM branch	64		Evaluation target	Labeled pixels
	PFM kernel sizes	11, 7, 3, 3, 3, 3		Output map	Saved
	PFM stride	2		GPU memory setting	Fixed fraction and growth enabled
	PFM padding	same			
	Activation	ReLU			
	Normalization	Batch normalization			

**Table 4:** Data categories and number of samples.

WHU-Hi-HongHu		Houston2013		Indian Pines	
Class	Samples	Class	Samples	Class	Samples
Re-ro	14,041	He-gr	1251	Alfalfa	46
Road	3512	St-gr	1254	Corn-notill	1428
Ba-so	21,821	Sy-gr	697	Corn-minti	830
Cotton	163,285	Trees	1244	Corn	237
Co-fi-wo	6218	Soil	1242	Gr-pasture	483
Rape	44,557	Water	325	Grass-trees	730
Ch-ca	24,103	Residential	1268	Gr-pa-mo	28
Pakchoi	4054	Commercial	1244	Hay-wi	478
Cabbage	10,819	Road	1252	Oats	20
Tu-mu	12,394	Highway	1227	So-no	972
Br-pa-en	11,015	Railway	1235	So-mi	2455
Br-ch	8954	Parking-1	1233	So-cl	593
Sm-br-ch	22,507	Parking-2	469	Wheat	205
La-sa	7356	Te-Co	428	Woods	1265
Celtuce	1002	Ru-Tr	660	Bu-G-Dr	386
Fil-co-le	7262			St-St-To	93
Ro-le	3010				
Carrot	3217				
Wh-ra	8712				
Ga-sp	3486				
Broad-be	1328				
Tree	4040				

**Table 5:** The comparative results on the WHU-Hi-HongHu dataset.

(**a**)											
**Train Sample**	**Class**	**Model**									
		**Lee**	**Hybrid**	**SVM_g**	**Vit**	**Cnn2D**	**He**	**Shar**	**FDSSC**	**DGPF**	**Ours**
50 per class	1	74.23%	94.23%	81.29%	90.29%	88.69%	73.29%	87.87%	87.06%	95.04%	**97.06%**
	2	8.96%	87.80%	9.08%	9.36%	85.11%	10.00%	9.85%	10.24%	92.74%	**98.05%**
	3	48.89%	77.91%	68.97%	73.42%	78.04%	75.55%	75.94%	78.35%	91.22%	**91.93%**
	4	42.24%	22.60%	76.73%	96.19%	65.61%	89.24%	95.53%	93.11%	95.28%	**97.41%**
	5	51.99%	68.68%	62.16%	91.88%	95.80%	77.06%	92.96%	97.80%	96.84%	**98.25%**
	6	5.92%	90.00%	75.66%	68.48%	87.86%	82.35%	87.67%	85.89%	95.85%	**97.32%**
	7	11.34%	56.64%	40.13%	9.55%	49.03%	38.75%	59.30%	64.71%	77.52%	**90.71%**
	8	51.37%	10.46%	31.27%	76.60%	49.83%	31.54%	67.71%	60.34%	92.55%	**97.58%**
	9	71.78%	**98.57%**	71.70%	67.65%	94.35%	75.45%	78.37%	80.63%	95.95%	98.15%
	10	31.27%	70.55%	38.10%	35.23%	70.69%	31.49%	73.97%	66.12%	85.83%	**93.57%**
	11	4.36%	38.22%	26.91%	43.90%	62.13%	19.34%	55.23%	69.36%	91.62%	**94.95%**
	12	41.45%	57.55%	46.21%	48.84%	17.77%	15.88%	68.01%	74.26%	82.48%	**91.35%**
	13	0.01%	49.84%	43.19%	7.99%	62.29%	60.04%	67.81%	63.26%	79.91%	**86.71%**
	14	60.81%	70.40%	50.22%	58.23%	72.45%	40.77%	73.82%	73.86%	91.99%	**95.93%**
	15	30.67%	86.87%	28.05%	34.66%	83.51%	36.87%	47.79%	50.63%	98.99%	**99.67%**
	16	26.38%	32.32%	56.33%	56.84%	72.75%	66.99%	72.41%	78.22%	97.02%	**97.72%**
	17	74.96%	74.86%	55.98%	80.41%	82.74%	35.74%	73.61%	76.93%	99.15%	**99.79%**
	18	62.36%	56.14%	42.97%	57.53%	91.73%	48.12%	74.36%	92.77%	**98.58%**	98.57%
	19	52.53%	59.64%	54.08%	50.76%	91.26%	56.73%	66.00%	74.30%	**92.19%**	96.08%
	20	62.51%	84.58%	47.85%	48.95%	78.90%	58.12%	66.73%	69.44%	97.58%	**98.83%**
	21	67.45%	47.26%	47.26%	48.20%	77.86%	50.31%	67.06%	68.78%	99.87%	**99.93%**
	22	0.69%	71.70%	61.05%	74.96%	91.45%	81.80%	85.86%	89.22%	98.74%	**99.46%**
	OA	34.74 ± 0.52%	49.78 ± 0.39%	64.84 ± 0.34%	70.66 ± 0.58%	70.47 ± 0.27%	71.56 ± 0.24%	82.95 ± 0.74%	82.79 ± 0.43%	92.43 ± 0.42%	**95.77 ± 0.19%**
	AA	42.54 ± 0.36%	63.95 ± 0.26%	50.69 ± 0.67%	55.91 ± 0.68%	74.99 ± 0.39%	52.52 ± 0.77%	70.36 ± 0.48%	72.97 ± 0.28%	93.04 ± 0.15%	**96.32 ± 0.08%**
	Kap	28.33 ± 0.38%	45.85 ± 0.47%	57.53 ± 0.21%	63.43 ± 0.37%	65.30 ± 0.28%	64.86 ± 0.18%	78.61 ± 0.49%	78.57 ± 0.55%	90.53 ± 0.35%	**94.67 ± 0.24%**
(**b**)											
**Train Sample**	**Class**	**Model**									
		**Lee**	**Hybrid**	**SVM_g**	**Vit**	**Cnn2D**	**He**	**Shar**	**FDSSC**	**DGPF**	**Ours**
25 per class	1	74.49%	75.43%	79.48%	82.06%	83.28%	73.14%	85.63%	86.08%	90.08%	**94.23%**
	2	8.89%	36.21%	8.62%	3.58%	7.86%	8.50%	11.14%	35.90%	87.96%	**91.61%**
	3	69.72%	66.96%	67.23%	62.03%	30.88%	74.77%	73.50%	71.80%	84.95%	**88.67%**
	4	4.73%	74.89%	71.76%	94.32%	91.04%	89.07%	94.23%	**97.69%**	91.52%	94.46%
	5	51.27%	71.35%	48.49%	87.86%	93.22%	70.21%	91.18%	93.67%	93.36%	**95.46%**
	6	16.68%	87.66%	70.79%	77.20%	91.83%	78.51%	88.17%	92.18%	92.16%	**93.11%**
	7	0	38.87%	35.97%	37.51%	57.41%	40.44%	45.55%	46.03%	71.82%	**78.93%**
	8	36.88%	61.78%	21.79%	15.74%	71.16%	17.13%	51.08%	71.36%	**84.85%**	80.11%
	9	78.08%	61.47%	70.80%	75.66%	79.43%	63.44%	78.66%	76.36%	94.33%	**95.71%**
	10	15.71%	77.49%	33.60%	44.55%	74.65%	17.36%	67.85%	68.83%	79.69%	**83.59%**
	11	3.28%	25.21%	21.43%	29.17%	55.61%	18.78%	56.84%	67.92%	80.42%	**88.47%**
	12	4.01%	47.21%	36.44%	30.33%	65.78%	32.59%	64.11%	70.28%	70.26%	**78.39%**
	13	0	46.91%	39.39%	53.29%	67.47%	50.94%	59.97%	63.70%	65.94%	**76.51%**
	14	47.72%	71.17%	45.23%	46.60%	69.27%	48.65%	67.44%	67.96%	82.99%	**88.21%**
	15	28.86%	13.82%	26.71%	12.38%	41.86%	42.17%	38.18%	51.48%	98.33%	**98.96%**
	16	32.63%	60.54%	50.06%	50.81%	68.14%	57.34%	67.80%	79.67%	93.64%	**94.36%**
	17	59.40%	93.53%	58.39%	68.68%	88.48%	79.10%	79.50%	83.92%	95.16%	**99.17%**
	18	30.01%	69.14%	39.41%	43.01%	84.12%	53.38%	80.89%	83.83%	97.72%	**98.37%**
	19	20.52%	47.33%	49.26%	24.67%	67.90%	42.18%	63.00%	69.33%	87.41%	**90.22%**
	20	39.87%	46.92%	43.57%	41.32%	72.72%	26.58%	64.11%	73.53%	96.38%	**97.96%**
	21	56.10%	42.21%	43.82%	65.31%	89.33%	43.05%	60.32%	71.53%	98.84%	**99.53%**
	22	37.93%	70.61%	55.59%	60.12%	87.67%	80.12%	87.32%	86.00%	93.65%	**96.53%**
	OA	18.19 ± 0.59%	67.84 ± 0.47%	60.18 ± 0.54%	72.00 ± 0.34%	79.06 ± 0.37%	69.54 ± 0.28%	80.27 ± 0.37%	83.87 ± 0.53%	87.24 ± 0.48%	**90.98 ± 0.32%**
	AA	32.58 ± 0.24%	58.49 ± 0.25%	46.27 ± 0.38%	50.28 ± 0.18%	69.96 ± 0.33%	50.34 ± 0.52%	67.11 ± 0.28%	73.14 ± 0.49%	87.79 ± 0.23%	**91.48 ± 0.17%**
	Kap	15.99 ± 0.28%	61.55 ± 0.63%	52.46 ± 0.49%	64.99 ± 0.29%	74.04 ± 0.28%	62.39 ± 0.46%	75.34 ± 0.48%	79.67 ± 0.35%	84.18 ± 0.56%	**88.74 ± 0.38%**
-	Para	**0.48 M**	1.88 M	-	2.73 M	4.61 M	7.67 M	2.29 M	1.61 M	5.15 M	5.22 M

Note: Bold values indicate the best result for each class or metric.

**Table 6:** The comparative results on the Houston2013 dataset.

Train Sample	Class	Model									
		Lee	Hybrid	SVM_g	Vit	Cnn2D	He	Shar	FDSSC	DGPF	Ours
50 per class	1	46.71%	95.19%	54.89%	96.04%	83.64%	32.46%	39.97%	61.89%	94.62%	**97.34%**
	2	54.72%	82.16%	43.22%	88.77%	**98.03%**	33.62%	46.57%	66.21%	96.18%	97.77%
	3	62.13%	93.04%	66.77%	88.10%	93.97%	69.55%	63.37%	66.77%	**99.88%**	99.64%
	4	0.00%	88.44%	24.46%	90.95%	90.20%	17.50%	32.08%	58.63%	96.28%	**98.29%**
	5	65.39%	95.28%	68.33%	83.99%	99.11%	61.57%	71.71%	86.39%	99.97%	**99.99%**
	6	22.55%	85.45%	22.55%	70.18%	90.18%	26.18%	32.36%	69.82%	99.16%	**99.29%**
	7	8.58%	52.37%	19.98%	76.52%	76.02%	12.41%	27.39%	46.05%	92.95%	**94.97%**
	8	7.35%	67.61%	27.61%	70.17%	74.19%	20.09%	52.05%	78.21%	86.35%	**92.84%**
	9	4.04%	68.04%	9.79%	70.62%	77.06%	10.48%	27.49%	54.90%	90.33%	**94.58%**
	10	32.54%	63.98%	44.77%	31.18%	80.29%	52.76%	68.14%	89.97%	97.08%	**98.59%**
	11	25.22%	61.10%	26.55%	69.36%	70.43%	1.69%	18.56%	49.47%	97.45%	**99.18%**
	12	14.03%	54.27%	27.90%	16.74%	78.95%	43.36%	39.81%	50.89%	93.88%	**96.62%**
	13	7.97%	87.44%	9.18%	42.51%	75.60%	2.66%	37.20%	7.73%	95.99%	**97.04%**
	14	47.88%	**100.00%**	41.27%	94.44%	95.77%	52.91%	56.08%	67.46%	99.97%	**100.00%**
	15	18.36%	92.62%	18.52%	89.67%	97.05%	10.82%	34.59%	39.67%	**100.00%**	**100.00%**
	OA	27.01 ± 0.28%	76.00 ± 0.34%	34.60 ± 0.43%	71.03 ± 0.35%	84.19 ± 0.30%	29.51 ± 0.28%	42.97 ± 0.54%	61.76 ± 0.23%	95.26 ± 0.21%	**97.37 ± 0.14%**
	AA	27.83 ± 0.56%	79.13 ± 0.57%	33.72 ± 0.37%	71.95 ± 0.24%	85.37 ± 0.34%	29.87 ± 0.24%	43.16 ± 0.32%	59.60 ± 0.14%	96.01 ± 0.35%	**97.74 ± 0.14%**
	Kappa	27.83 ± 0.45%	74.10 ± 0.27%	29.23 ± 0.12%	68.71 ± 0.39%	82.89 ± 0.52%	24.44 ± 0.45%	38.39 ± 0.33%	58.57 ± 0.35%	94.87 ± 0.11%	**97.16 ±** **0.15%**
-	Params	**3.19 M**	1.87 M	-	2.58 M	4.59 M	2.66 M	2.25 M	0.85 M	3.49 M	3.55 M

Note: Bold values indicate the best result for each class or metric.

**Table 7:** The comparative results on the Indian Pines dataset.

Train Sample	Class	Model									
		Lee	Hybrid	SVM_g	Vit	Cnn2D	He	Shar	FDSSC	DGPF	Ours
5%	1	0.00%	0.00%	0.00%	0.00%	0.00%	0.00%	0.00%	0.00%	67.50%	**95.24%**
	2	30.62%	33.21%	22.09%	0.30%	58.71%	19.05%	32.10%	50.93%	91.49%	**95.74%**
	3	0.00%	4.45%	8.54%	41.42%	54.98%	0.36%	5.34%	17.79%	92.50%	**94.04%**
	4	53.95%	87.50%	49.34%	91.18%	90.79%	35.53%	80.92%	86.18%	92.52%	**95.39%**
	5	30.12%	47.89%	30.72%	77.57%	75.30%	33.13%	26.81%	33.43%	93.75%	**93.86%**
	6	0.00%	0.00%	3.02%	83.24%	23.46%	0.00%	4.94%	1.10%	96.41%	**97.01%**
	7	0.00%	**100.00%**	0.00%	0.00%	**100.00%**	0.00%	0.00%	0.00%	66.30%	95.38%
	8	0.00%	0.00%	0.26%	75.65%	17.63%	0.00%	10.26%	0.00%	99.58%	**99.96%**
	9	0.00%	0.00%	0.00%	0.00%	0.00%	0.00%	0.00%	0.00%	68.95%	**87.78%**
	10	44.25%	92.69%	45.20%	28.52%	77.40%	59.27%	50.61%	62.65%	91.92%	**94.91%**
	11	0.00%	0.89%	31.77%	78.91%	59.92%	16.53%	46.83%	57.42%	95.78%	**96.69%**
	12	0.00%	11.16%	23.12%	21.65%	84.38%	1.83%	21.91%	43.41%	90.35%	**93.63%**
	13	1.41%	**100.00%**	3.52%	69.68%	98.59%	0.00%	0.00%	52.82%	97.23%	**96.98%**
	14	0.53%	81.74%	60.73%	21.02%	96.37%	46.63%	63.39%	63.74%	**99.07%**	98.97%
	15	6.21%	0.00%	7.45%	33.56%	50.31%	0.00%	13.66%	34.78%	**97.38%**	94.30%
	16	0.00%	51.69%	0.00%	79.07%	85.39%	0.00%	0.00%	2.25%	79.10%	**86.05%**
	OA	11.06 ± 0.45%	31.06 ± 0.29%	28.32 ± 0.47%	47.40 ± 0.49%	64.07 ± 0.50%	20.78 ± 0.18%	35.50 ± 0.20%	45.31 ± 0.23%	94.37 ± 0.11%	**96.09 ± 0.11%**
	AA	11.93 ± 0.24%	43.66 ± 0.38%	20.41 ± 0.25%	53.98 ± 0.12%	69.52 ± 0.22%	15.17 ± 0.54%	25.48 ± 0.38%	36.18 ± 0.19%	88.74 ± 0.30%	**94.75 ± 0.20%**
	Kappa	4.43 ± 0.16%	25.21 ± 0.27%	20.16 ± 0.28%	38.61 ± 0.37%	59.44 ± 0.48%	13.26 ± 0.36%	26.88 ± 0.18%	37.68 ± 0.29%	93.58 ± 0.26%	**95.54 ± 0.13%**
-	Params	**0.39 M**	1.88 M	-	2.65 M	4.60 M	4.08 M	2.26 M	1.19 M	4.23 M	4.29 M

Note: Bold values indicate the best result for each class or metric.

**Table 8:** Data categories and number of samples on Xiongan and OHID-1.

Xiongan		OHID-1	
Class	Samples	Class	Samples
Background	2,247,890	Building	661,721
Residential	225,647	Farmland	438,849
Commercial	180,766	Forest	370,901
Industrial	15,353	Road	129,648
Road	452,144	Water	598,694
Water	475,591	Bareland	14,699
Green space	169,342	Fishpond	43,846
Agricultural	23,304		
Bare soil	165,647		
Construction	38,409		
Park	193,830		
Forest	5612		
Grassland	59,165		
Wetland	1,026,513		
Farmland	7151		
Orchard	91,072		
Vineyard	29,148		
Facil agri	1496		
Building	421,790		
Structure	65,514		
Other	29,616		

**Table 9:** Per-class and overall results on OHID-1 under two training budgets (50 samples per class and 1%).

Train Sample	Class	DGPF	Ours	Train Sample	Class	DGPF	Ours
50	1	45.94%	**73.29%**	1%	1	77.74%	**94.34%**
	2	61.73%	**92.79%**		2	**95.99%**	94.00%
	3	71.57%	**83.65%**		3	88.87%	**96.30%**
	4	46.69%	**61.51%**		4	65.92%	**73.90%**
	5	74.08%	**80.67%**		5	83.79%	**95.10%**
	6	92.20%	**100.00%**		6	**100.00%**	90.21%
	7	90.10%	**90.38%**		7	92.37%	**93.78%**
	OA	59.65 ± 1.09%	**80.93 ± 0.45%**		OA	84.60 ± 0.11%	**93.59 ± 0.08%**
	AA	68.91 ± 0.55%	**83.19 ± 0.46%**		AA	86.38 ± 0.13%	**91.09 ± 0.09%**
	Kappa	49.02 ± 1.15%	**73.39 ± 0.55%**		Kappa	78.47 ± 0.15%	**91.72 ± 0.11%**
	Params	**2.01 M**	2.08 M		Params	**2.01 M**	2.08 M

Note: Bold values indicate the best result for each class or metric.

**Table 10:** Per-class and overall results on Xiongan under two training budgets (50 samples per class and 1%).

Train Sample	Class	DGPF	Ours	Train Sample	Class	DGPF	Ours
50	1	**100.00%**	**100.00%**	1%	1	100.00%	**100.00%**
	2	52.10%	**81.19%**		2	**99.89%**	99.59%
	3	54.54%	**80.59%**		3	99.80%	**99.99%**
	4	75.03%	**98.87%**		4	99.98%	**100.00%**
	5	86.88%	**97.65%**		5	97.18%	**99.22%**
	6	43.04%	**64.72%**		6	99.25%	**99.96%**
	7	59.57%	**94.99%**		7	99.75%	**99.86%**
	8	81.37%	**98.15%**		8	99.99%	**100.00%**
	9	88.43%	**95.85%**		9	97.89%	**99.62%**
	10	94.30%	**99.10%**		10	99.96%	**100.00%**
	11	82.45%	**99.25%**		11	99.25%	**99.89%**
	12	66.56%	**98.55%**		12	**100.00%**	**100.00%**
	13	59.29%	**90.45%**		13	85.40%	**94.62%**
	14	39.62%	**58.71%**		14	84.58%	**95.47%**
	15	82.44%	**98.52%**		15	**100.00%**	**100.00%**
	16	66.24%	**94.74%**		16	76.97%	**91.52%**
	17	58.31%	**83.44%**		17	98.52%	**99.96%**
	18	82.93%	**88.52%**		18	99.89%	**100.00%**
	19	49.71%	**84.65%**		19	58.98%	**64.17%**
	20	67.82%	**96.81%**		20	99.96%	**99.99%**
	21	84.83%	**97.43%**		21	97.19%	**99.70%**
	OA	57.02 ± 1.52%	**81.73 ± 0.86%**		OA	85.89 ± 0.11%	**93.77 ± 0.08%**
	AA	70.26 ± 1.24%	**89.58 ± 1.13%**		AA	93.55 ± 0.16%	**97.31 ± 0.03%**
	Kappa	52.47 ± 2.36%	**80.13 ± 1.26%**		Kappa	83.73 ± 0.13%	**92.68 ± 0.09%**
	Params	**4.97 M**	5.03 M		Params	**4.97 M**	5.03 M

Note: Bold values indicate the best result for each class or metric.

**Table 11:** Averaged OA, AA, and Kappa across benchmark settings. Each metric reports the mean value and the difference to EMNet in percentage points.

Method	OA (%) Δ to EMNet	AA (%) Δ to EMNet	Kappa (%) Δ to EMNet
EMNet	96.41% Δ +0.00	96.27% Δ +0.00	95.79% Δ +0.00
DGPF	94.02% Δ −2.39	92.60% Δ −3.67	92.99% Δ −2.80
Cnn2D	72.91% Δ −23.50	76.63% Δ −19.64	69.21% Δ −26.58
FDSSC	63.29% Δ −33.12	56.25% Δ −40.02	58.27% Δ −37.52
Vit	63.03% Δ −33.38	60.61% Δ −35.66	56.92% Δ −38.87
Sharma	53.81% Δ −42.60	46.33% Δ −49.94	47.96% Δ −47.83
Hybrid	52.28% Δ −44.13	62.25% Δ −34.02	48.39% Δ −47.40
SVM_g	42.59% Δ −53.82	34.94% Δ −61.33	35.64% Δ −60.15
He	40.62% Δ −55.79	32.52% Δ −63.75	34.19% Δ −61.60
Lee	24.27% Δ −72.14	27.43% Δ −68.84	20.20% Δ −75.59

**Table 12:** Q statistics between EMNet and compared methods across benchmarks using class-wise median discretization.

Method	WHU-Hi-HongHu	Houston2013	Indian Pines	Weighted Avg.
EMNet	1.000	1.000	1.000	1.000
DGPF	0.906	1.000	0.800	0.901
Cnn2D	0.753	0.765	0.000	0.529
FDSSC	0.180	0.765	0.471	0.433
Vit	0.180	0.379	0.471	0.324
Sharma	−0.180	0.379	0.000	0.032
Hybrid	0.180	0.379	0.471	0.324
SVM_g	−0.180	0.379	0.000	0.032
He	−0.180	0.379	0.000	0.032
Lee	0.508	0.765	0.250	0.503

**Table 13:** Average ranks of compared methods based on OA over four evaluation settings from the Friedman test.

Method	EMNet	DGPF	Cnn2D	FDSSC	Shar	Vit	Hybrid	He	SVM_g	Lee
Rank	1	2	3	4	5	6	7	8	9	10
Average Rank	1.00	2.00	4.50	4.50	5.00	5.25	7.00	7.50	8.25	10.00

**Table 14:** 95% confidence intervals and Cohen’s d effect sizes for OA improvements over 10 random splits.

Dataset	Budget	DGPF OA	DGPF CI	EMNet OA	EMNet CI	ΔOA	Cohen’s d
OHID-1	50	59.65 ± 1.09%	[58.87, 60.43]	80.93 ± 0.45%	[80.61, 81.25]	21.28 pp	25.52
OHID-1	1%	84.60 ± 0.11%	[84.52, 84.68]	93.59 ± 0.08%	[93.53, 93.65]	8.99 pp	93.47
Xiongan	50	57.02 ± 1.52%	[55.93, 58.11]	81.73 ± 0.86%	[81.11, 82.35]	24.71 pp	20.01
Xiongan	1%	85.89 ± 0.11%	[85.81, 85.97]	93.77 ± 0.08%	[93.71, 93.83]	7.88 pp	81.93

**Table 15:** Overall ablation results on the WHU-Hi-HongHu dataset.

	OA	AA	Kappa
DGPF-RENet(baseline)	92.43%	93.04%	90.53%
DGPF-RENet + EGM	92.71%	93.14%	90.86%
DGPF-RENet + CFC	92.41%	93.12%	90.51%
DGPF-RENet + EGM + CFC	**95.77%**	**96.32%**	**94.67%**

Note: Bold values indicate the best result for each class or metric.

**Table 16:** Component-wise ablation of EMG based on the CFC baseline.

	OA	AA	Kappa
CFC	92.41%	93.12%	90.51%
CFC + EMG_noAttnFuse	92.63%	93.22%	90.77%
CFC + EMG_noTransform	92.82%	93.29%	91.00%
CFC + EMG_noAttnFuse_noTransform	92.64%	93.16%	90.78%
CFC + EMG	**95.77%**	**96.32%**	**94.67%**

Note: Bold values indicate the best result for each class or metric.

**Table 17:** CFC ablation based on the EMG baseline.

	OA	AA	Kappa
EMG	92.71%	93.14%	90.86%
EMG + CFC(1)	92.92%	93.26%	91.12%
EMG + CFC(2)	**95.77%**	**96.32%**	**94.67%**
EMG + CFC(1) + (2)	92.83%	93.24%	91.01%

Note: Bold values indicate the best result for each class or metric.

**Table 18:** Preliminary experiment on Indian Pines under synthetic rain and snow perturbations.

	Origin		Rain		Snow	
	DGPF	Ours	DGPF	Ours	DGPF	Ours
OA	94.37 ± 0.11%	96.09 ± 0.11%	88.46 ± 0.16%	94.90 ± 0.20%	82.78 ± 1.03%	93.73 ± 0.17%
AA	88.74 ± 0.30%	94.75 ± 0.20%	76.84 ± 0.58%	90.83 ± 0.68%	67.64 ± 1.16%	88.73 ± 0.21%
Kappa	93.58 ± 0.26%	95.54 ± 0.13%	86.88 ± 0.17%	94.19 ± 0.23%	80.41 ± 1.18%	92.86 ± 0.19%

## Data Availability

Public hyperspectral datasets WHU-Hi-HongHu, Houston2013, Indian Pines, OHID-1, and Xiongan (Xiongan New Area) were analyzed in this study. All datasets are openly available from their original providers. No new data were created in this work.

## Abbreviations

The following abbreviations are used in this manuscript:

HSI	Hyperspectral image
EMNet	Equivariant Manifold Network
EGM	Equivariance-Guided Module
CFC	Characteristic Filtering Convolution
DGA	Decoupled gaze attention
REM	Redundancy elimination module
PFM	Phantom fractal module
SE(2)	Special Euclidean group in 2D
HiT	Hyperspectral Image Transformer
LeViT	Lightweight vision transformer architecture
FLOPs	Floating-point operations
ANN	Artificial neural network
GAN	Generative adversarial network
CNN	Convolutional neural network
DCNN	Deep convolutional neural network
